# Isolation and molecular identification of nematode surface mutants with resistance to bacterial pathogens

**DOI:** 10.1093/g3journal/jkad056

**Published:** 2023-03-13

**Authors:** Delia O’Rourke, Maria J Gravato-Nobre, Dave Stroud, Emily Pritchett, Emily Barker, Rebecca L Price, Sarah A Robinson, Simon Spiro, Patricia Kuwabara, Jonathan Hodgkin

**Affiliations:** Department of Biochemistry, University of Oxford, Oxford OX1 3QU, UK; Department of Biochemistry, University of Oxford, Oxford OX1 3QU, UK; Department of Biochemistry, University of Oxford, Oxford OX1 3QU, UK; Department of Biochemistry, University of Oxford, Oxford OX1 3QU, UK; Department of Biochemistry, University of Oxford, Oxford OX1 3QU, UK; Department of Biochemistry, University of Oxford, Oxford OX1 3QU, UK; Department of Biochemistry, University of Oxford, Oxford OX1 3QU, UK; Department of Biochemistry, University of Oxford, Oxford OX1 3QU, UK; School of Biochemistry, University of Bristol, Bristol BS8 1TD, UK; Department of Biochemistry, University of Oxford, Oxford OX1 3QU, UK

**Keywords:** *C. elegans*, nematode pathogens, surface genetics, bacterial resistance, GT92, glycosyltransferase, Catel–Manzke syndrome

## Abstract

Numerous mutants of the nematode *Caenorhabditis elegans* with surface abnormalities have been isolated by utilizing their resistance to a variety of bacterial pathogens (*Microbacterium nematophilum*, *Yersinia pseudotuberculosis*, and 2 Leucobacter strains), all of which are able to cause disease or death when worms are grown on bacterial lawns containing these pathogens. Previous work led to the identification of 9 *srf* or *bus* genes; here, we report molecular identification and characterization of a further 10 surface-affecting genes. Three of these were found to encode factors implicated in glycosylation (*srf-2*, *bus-5*, and *bus-22*), like several of those previously reported; *srf-2* belongs to the GT92 family of putative galactosyltransferases, and *bus-5* is homologous to human dTDP-D-glucose 4,6-dehydratase, which is implicated in Catel–Manzke syndrome. Other genes encoded proteins with sequence similarity to phosphatidylinositol phosphatases (*bus-6*), Patched-related receptors (*ptr-15/bus-13*), steroid dehydrogenases (*dhs-5/bus-21*), or glypiation factors (*bus-24*). Three genes appeared to be nematode-specific (*srf-5*, *bus-10*, and *bus-28*). Many mutants exhibited cuticle fragility as revealed by bleach and detergent sensitivity; this fragility was correlated with increased drug sensitivity, as well as with abnormal skiddy locomotion. Most of the genes examined were found to be expressed in epidermal seam cells, which appear to be important for synthesizing nematode surface coat. The results reveal the genetic and biochemical complexity of this critical surface layer, and provide new tools for its analysis.

## Introduction

The surface of the nematode *Caenorhabditis elegans* constitutes the main interface between this organism and its surrounding milieu. Consequently, it has major functions as a permeability barrier and as the main area of tractional contact for vermiform locomotion. It also provides the site of attack for a multitude of bacterial and fungal pathogens, which must be able to recognize and bind to the worm's surface in order to initiate infection. The nematode epidermis (hypodermis) has a complex structure and constitutes a defining characteristic of the phylum Nematoda (Page and Johnston 2007). Its main extracellular layer consists of a thick collagenous cuticle, which has important mechanical functions in providing rigidity and maintenance of a hydrostatic skeleton; this is surrounded by an epicuticle and surface coat. The detailed biochemistry of these layers remains uncertain. All must be synthesized in a cyclical manner during the growth of the worm, starting with embryogenesis and then at each of 4 successive larval molts (Lažetić and Fay 2017). The main ectodermal tissue, the hypodermal syncytium, is believed to provide most or all of the cuticular collagens, while a set of specialized cells, the seam cells, which run along the lateral midlines, may be more important for providing surface coat. However, little is certain about the detailed cell biology of nematode molting (Singh and Sulston 1978; Lažetić and Fay 2017).

**Fig. 1.**
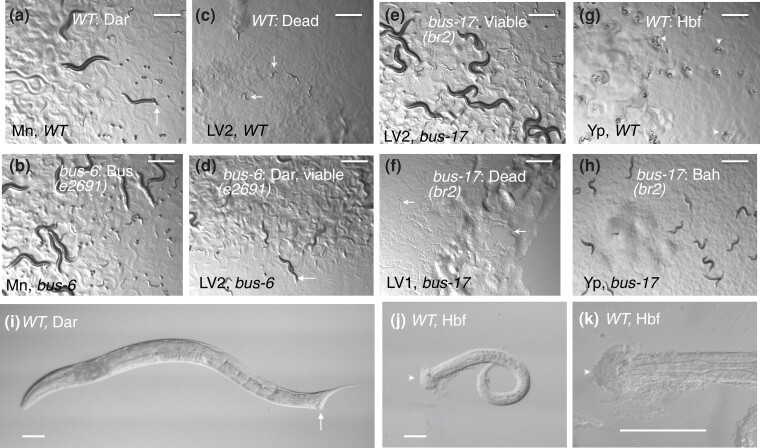
Pathogenic responses. Panels a) to f) show progeny of 5 adult hermaphrodites after 3 days of growth on pathogenic lawns; panels g) and h) show larvae, 24 h after hatching on *Yersinia* lawns; panels i) to k) show higher magnification images of Dar adult (i) or Hbf (j, k) L1 worms. a), b) Worms growing on *E. coli/M. nematophilum* lawns. a) Wild type: poor growth, Dar phenotype; (b) *bus-6*: vigorous growth; Bus phenotype c) to e), Worms growing on *E. coli/Leucobacter* Verde2 lawns. c) Wild type (inviable with larval death); (d) *bus-6* (poorly viable, weak Dar phenotype); e) *bus-17* (vigorous growth, Bus phenotype); (f) *bus-17* growing on *E. coli*/*Leucobacter* Verde1 (early larval death); (g, h) Worms growing on lawns of YPIII. g) Wild type with larval Head BioFilm (Hbf phenotype); (h) *bus-17*: Biofilm Absent on Head (Bah phenotype). Arrows indicate tail swelling in a), d), i); barbed arrows indicate dead larvae in c), f); arrowheads indicate head biofilm in e), j), and k). Scale bars 500 microns a)–h), 50 microns i)–k). Pathogens: Mn, *M. nematophilum*; LV1, *Leucobacter* Verde1; LV2, *Leucobacter* Verde2; Yp, *Y. pseudotuberculosis* YPIII.

Genetic approaches to the investigation of the *C. elegans* surface were initiated by the isolation of a series of *srf* (SuRFace abnormal) mutants defined by alterations in antigenicity or lectin binding (Politz *et al*. 1990; Link *et al*. 1992). More recently, bacterial pathogens that attack by attaching to the nematode surface have provided an efficient means of screening or selecting for mutants with surface alterations that prevent infection. *Microbacterium nematophilum* attaches to the rectal epithelium and elicits a conspicuous tail swelling, the Dar or Deformed Anal Region phenotype (Hodgkin *et al*. 2000). Mutants that fail to exhibit this swelling response define a set of at least 20 *bus* (Bacterially Un-Swollen) genes (Fig. 1, a and b; Gravato-Nobre *et al*. 2005). Most *bus* mutants are altered in primary infection, failing to support the formation of a rectal colony of bacteria, rather than being defective in the cellular swelling, which appears to be a defensive inflammatory response (Nicholas and Hodgkin 2004). Many of the uninfectable *bus* mutants have demonstrable alterations in general surface properties (Gravato-Nobre *et al*. 2005, 2011; Yook and Hodgkin 2007).

A different surface infection paradigm is provided by *Yersinia* bacteria: larval worms grown on lawns of the bacterium *Yersinia pseudotuberculosis* develop a cap of bacterial biofilm on their heads (Hbf or Head BioFilm phenotype), and mutants that fail to accumulate biofilm define a set of *bah* (Biofilm Absent on Head) mutants (Darby *et al*. 2007; Fig. 1, e and f). There is a substantial overlap between mutants in these classes—thus, *srf-2*, *srf-3*, and *srf-5* mutants all exhibit Srf, Bus, and Bah phenotypes (Darby *et al*. 2007). This overlap is further discussed in the present paper.

Two complementary pathogens have provided powerful tools for selecting additional mutants with surface alteration. Two bacterial strains belonging to the genus *Leucobacter* were isolated from a coinfected *Caenorhabditis tropicalis* nematode obtained from Cape Verde (*Leucobacter celer astrifaciens*, known as Verde1 and *Leucobacter musarum musarum*, known as Verde2; Hodgkin *et al*. 2013; Clark and Hodgkin 2015). Verde2 induces tail swelling in *C. elegans*, like *M. nematophilum*, which is accompanied by lethal infection, so mutants that are resistant to Verde2 killing can be directly selected from large mutagenized populations. Most of the previously described *bus* mutants, plus others reported in this paper, are fully resistant to Verde2. A few are able to survive on Verde2 lawns but grow poorly and exhibit the tail-swelling response (Dar or Deformed Anal Region phenotype; Fig. 1, b and c). In contrast, Verde1 bacteria are able to attach to the worm's surface but do not kill wild-type *C. elegans* except through the formation of worm stars when worms are swimming in liquid (Hodgkin *et al*. 2013). However, Verde1 bacteria efficiently kill most Verde2-resistant mutants when worms are grown on bacterial lawns containing Verde1, unlike wild-type worms, which survive and can reproduce on Verde1 lawns. A different class of surface-abnormal-resistant mutants has, therefore, been selected by mutagenizing various *srf* and *bus* mutants and selecting for their survival on Verde1 bacterial lawns (Loer *et al*. 2015).

These bacterial pathogens also provide convenient tools for phenotyping and have thereby facilitated the molecular identification of surface-abnormal mutants. Previous work characterized *srf-3* (Höflich et al. 2004), *bus-17* and *bus-19* (Yook and Hodgkin 2007), *bus-8* (Partridge *et al*. 2008), *bus-1* and *bus-18* (Gravato-Nobre and Hodgkin 2008), *bah-1* (Drace *et al*. 2009), and *bus-2*, *bus-4*, and *bus-12* (Gravato-Nobre *et al*. 2011). Most of these 9 genes were found to encode proteins with sequence implicating them in protein glycosylation, and in some cases, glycan alterations have been demonstrated in the corresponding mutants (Cipollo *et al*. 2004; Palaima *et al*. 2010; Parsons *et al*. 2014).

In this report, we expand the set of mutants with surface alterations that result in bacterial resistance and describe the molecular identification and more detailed characterization of 10 genes: *srf-2*, *srf-5*, *bus-5*, *bus-6*, *bus-10*, *bus-13*, *bus-21*, *bus-22*, *bus-24*, and *bus-28*. Several *bah* genes, in addition to *bah-1* (Drace *et al*. 2009), have now also been defined at a molecular level and will be described elsewhere (O’Rourke *et al.*, in preparation).

Genes affecting sensitivity to *Leucobacter* Verde1 represent a complementary set to the *srf*, *bus*, and *bah* genes; some of these have been reported already (Loer *et al*. 2015) and others will be described in a separate publication (O’Rourke *et al.*, in preparation).

Cloning of the 10 genes discussed in the present paper was mostly enabled by the availability of whole genome sequencing (Sarin *et al*. 2008) and detailed high-quality genome annotation (Davis *et al*. 2022). The results expand the *C. elegans* roster of functional glycosylation factors and enigmatic nematode-specific proteins, some of which can now be assigned biological functions.

## Materials and methods

### Culture and infection methods

General methods for *C. elegans* culture, manipulation, microscopy, ethyl methanesulfonate (EMS) mutagenesis, and pathogen infection were as described previously (Brenner 1974; Gravato-Nobre *et al*. 2005, 2011; Hodgkin *et al*. 2013). Most resistance selections and assays were carried out at 25°C, using mixed bacterial lawns (90% *E. coli* OP50, 10% pathogen). Assays for *Yersinia* biofilm accumulation on larvae were carried out as described previously (Darby *et al*. 2007) using spots of LB-grown *Yersinia pseudotuberculosis* strain YPIII on NGM agar, incubated for 24 h at 25°. Eggs or gravid adults were added to the spots and larval hatchlings were examined for head biofilm accumulation after a further 24–28 h (Fig. 1, g and h). Strains are listed in Supplementary Materials.

### Bleach sensitivity assay

Essentially as in Gravato-Nobre *et al*. (2005): for each test, a 20-ml drop of alkaline hypochlorite solution [1 N NaOH, 40% NaOCl solution (∼12% available chlorine)] was placed on NGM agar, and 15 adult hermaphrodites were immediately transferred to the drop, using a platinum wire worm pick. The time in seconds taken before all the worms stopped thrashing and the time taken for the first worms to break up were noted.

**Table:** 

Rank	Time for all worms to stop moving (s)	Time for the first worms to break up (s)
+	> 20	>100
++	< 20	<100
+++	< 15	< 50
++++	<5	< 20

### Movement assays

Crawling mobility (distance crawled on a bacterial lawn) was measured as in Matthews *et al*. (2021): 15 adult hermaphrodites were placed on one end of a 50 × 5 mm lawn of *E. coli* OP50, and the distance moved by the 10 fastest animals was measured after 10 min. Relative mobility was expressed as a percentage ± SD of N2 wild type. Liquid thrashing rates were measured by placing a single adult hermaphrodite in a 20-μl drop of M9 buffer on NGM agar, waiting 30 s for equilibration, then counting the number of thrashes in the next 60 s; 5 −10 individuals were measured for each genotype. Movement assays were carried out at 22°C.

### Molecular methods

Reporter gene constructs were generated as in Hobert (2002). The CRISPR/Cas9 deletion for *bus-5* was generated as in Farboud and Meyer (2015), with details in Supplementary Materials, using gRNA exon 1: 5′ GACAUGCGUUCUGAUAACUGGGUUUUAGAGCUGUUUUG and gRNA exon 5: 5′GAUAUGUGGAAGACUGCUCGGGUUUUAGAGCUAUGCUGUUUUG.

## Results

### Isolation of bacterial resistance mutants

Previous searches for *C. elegans* mutants resistant to *M. nematophilum* infection were carried out by visual screening of mutagenized populations exposed to this pathogen, looking for the absence of the conspicuous tail swelling induced by this infection. The lethal infection caused by *Leucobacter* Verde2 (Hodgkin *et al*. 2013) allowed a more direct selection of resistant mutants: wild-type worms were mutagenized with EMS, grown for one generation on *E. coli* OP50 and then washed onto *E. coli/*Verde2-mixed bacterial lawns. After 2 generations of further growth, healthy survivors were picked and established new mutant lines. All proved to be stable mutants with resistance to both *Leucobacter* Verde2 and *M. nematophilum.* From several such selections, further isolates of many of the known *bus* loci (*srf-2*, *srf-5*, *bus-2*, *bus-4*, *bus-5*, and *bus-8*) were recovered (Supplementary Table 1). In addition, mutants defining new *bus* loci were obtained: *bus-22* and *bus-24* are described below. Further mutants, defining at least 3 distinct sex-linked loci, were obtained but have not been analyzed further. A mutant of one new *bus* gene, *bus-28*, was obtained from the Million Mutation Project (Thompson *et al*. 2013).

Mutant growth on 4 bacterial substrates (mixed lawns of *E. coli* plus *M. nematophilum*, *Leucobacter* Verde1 or Verde2, and pure *Y. pseudotuberculosis* YPIII) was examined and is summarized in Table 1 for representative alleles of 19 genes for which molecular identities have been determined (9 in previous reports, 10 in this paper). Typical phenotypes are shown in Fig. 1. All mutants grew well on lawns of pure *E. coli* OP50, although some exhibited detectable abnormalities, such as small size, slow growth, abnormal skiddy movement (Skd), and some cuticle fragility, manifested by hypersensitivity to bleach and detergent (Supplementary Methods). These phenotypes are summarized in Table 2.

**Table 1.** Responses to pathogens.

Gene	Alleles	Phenotypes on pathogenic bacterial lawns			
		*M. nem.*	*Leucobacter*	*Leucobacter*	*Y.pseudo.*
			Verde2	Verde1	YPIII
WT(N2)	NA	Dar	Dead	Skd	Hbf
*srf-2*	23	Bus	Viable	Dead	Bah
*srf-3*	5	Bus	Viable	Dead	Bah
*srf-5*	4	Bus	Viable	Dead	Bah
*bus-1*	24	Bus	Dar	Skd	Hbf
*bus-2*	9	Bus	Viable	Dead	Bah
*bus-4*	10	Bus	Viable	Dead	Bah
*bus-5*	18	Bus	Viable	Dead	Bah
*bus-6*	7	Bus	Dar	Gro, Skd	Weak Bah
*bus-8*	11	Bus	Viable	Dead	Hbf
*bus-10*	33	Bus	Viable	Dead	Weak Bah
*bus-12*	9	Bus	Viable	Dead	Bah
*bus-13*	1	Bus	Dead	Dead	Hbf
*bus-17*	5	Bus	Viable	Dead	Bah
*bus-18*	1	Bus	Viable	Gro, Skd	Hbf
*bus-19*	3	Bus	Viable	Dead	Hbf
*bus-21*	3	Bus	Viable	Dead	Bah
*bus-22*	3	Bus	Viable	Dead	Bah
*bus-24*	4	Bus	Viable	Gro, Skd	Hbf
*bus-28*	1	Bus	Dar	Gro, Skd	Hbf

The columns report the number of independent alleles, followed by phenotypes (survival, anal swelling, biofilm) for a severe mutant allele of each gene, usually a null. Exceptions are *bus-8* and *bus-13*, null alleles of which are inviable so mutants could not be tested. Alleles used were: *srf-2(yj262)*, *srf-3(yj10)*, *srf-5(ct115)*, *bus-1(e2678)*, *bus-2(e2687)*, *bus-4(br4)*, *bus-5(br19)*, *bus-6(e2691)*, *bus-8(e2883)*, *bus-10(e2702)*, *bus-12(e2977)*, *bus-13(e2710)*, *bus-17(br2)*, *bus-18(e2795)*, *bus-19(e2912)*, *bus-21(e2997)*, *bus-22(e2798)*, *bus-24(e3020)*, *bus-28(gk236264)*. Abbreviations: *M. nem*, *M. nematophilum*; *Y. pseudo*, *Y. pseudotuberculosis*; WT, wild type; NA, not applicable. Phenotype abbreviations: Dar, deformed anal region; Bus, bacterially unswollen; Gro, slow growth; Skd, skiddy locomotion; Bah, biofilm absent on head; Hbf, head biofilm present.

**Table 2.** Nonpathogenic phenotypes.

Gene	Morphology	25°g.t.	Mobility%	Bleach sensitivity
WT(N2)	WT	58	100 ± 3	+
*srf-2*	WT	61	57 ± 4 S	++
*srf-3*	WT	66	60 ± 3 S	+++
*srf-5*	WT	64	76 ± 7	+
*bus-1*	WT	62	77 ± 5	+
*bus-2*	WT	63	89 ± 4	++
*bus-4*	WT	63	68 ± 2	+ +
*bus-5*	WT	68	17 ± 2 SS	++++
*bus-6*	WT	63	64 ± 1	+
*bus-8*	Sma	77	17 ± 1 SS	++++
*bus-10*	WT	64	77 ± 4	+
*bus-12*	WT	63	88 ± 2	++
*bus-13*	WT	63	71 ± 4	+++
*bus-17*	WT	66	60 ± 3 S	++++
*bus-18*	WT	67	41 ± 3 S	+++
*bus-19*	Sma	78	16 ± 2 SS	++++
*bus-21*	Sma	77	10 ± 1 SS	++++
*bus-22*	WT	62	80 ± 2	++
*bus-24*	Sma	71	21 ± 1 SS	++++
*bus-28*	WT	62	81 ± 3	+

Mutant alleles as in Table 1. Generation time (25° g.t) was measured in hours from egg-hatch to first progeny egg-hatch, mean of 2–5 measurements at 25°C.

Mobility (distance crawled on a bacterial lawn) was measured as in Mathews *et al*. (2021), expressed as a percentage ± SD of N2 wild type. Bleach sensitivity was assessed as in Gravato-Nobre *et al*. (2005) and explained in Materials and Methods. S, slight Skd; SS, severe Skd.

All mutants exhibited a robust Bus phenotype, that is the absence of rectal swelling and unimpaired development, when grown on lawns containing *M. nematophilum*. As previously reported (Gravato-Nobre *et al*. 2005; Yook and Hodgkin 2007), little or no rectal colonization by this pathogen was observed for most of these mutants; however, *bus-21*, *bus-22*, *bus-24*, and *bus-28* have not been examined by vital staining. Previously, we reported mutants with rectal colonization but no cellular swelling, such as *sur-2* and *egl-5* (Nicholas and Hodgkin 2004, 2009), but no new mutants of this type were obtained. A *sur-2* mutant originally isolated as *bus-9(e2706)* (Gravato-Nobre *et al*. 2005) was found to die rapidly without rectal swelling on lawns containing *Leucobacter* Verde2. This observation suggested that such mutants could not be isolated by selection on Verde2 lawns.

The mutants’ Bus response to *M. nematophilum* was strongly though not completely correlated with resistance to *Leucobacter* Verde2, which is lethal to wild-type worms. Fifteen mutants grew well in the presence of Verde2, with no sign of infection, and another 3 were able to survive but grew poorly, usually with a Dar phenotype (Fig. 1c). The partial resistance exhibited by *bus-1* mutants suggested that the rectal epithelia, where *bus-1* is expressed (Gravato-Nobre and Hodgkin 2008), provide a major route for infection by Verde2, but not the only route. Exceptionally, the *bus-13* mutant was killed by both Verde1 and Verde2.

Sensitivity to *Leucobacter* Verde1 was mostly correlated with resistance to Verde2. Twelve mutants were fully resistant to Verde2 but killed by Verde1. Three were resistant to Verde2 and survived poorly on Verde1 (*bus-18*, *bus-22*, and *bus-24*). Three survived but grew poorly on both pathogens (*bus-1*, *bus-6*, and *bus-28*).

Resistance to *Yersinia* biofilm formation (Bah phenotype) was also examined: 10 of the 19 mutants were fully Bah when larvae developed on *Yersinia* (YPIII) lawns, and 2 more showed a weak Bah phenotype, with significantly less accumulation of biofilm. Surprisingly, viable *bus-8* mutants (Partridge *et al*. 2008) accumulated biofilm-like wild type, as did several other mutants (*bus-1*, *bus-13*, *bus-18*, *bus-19*, *bus-24*, and *bus-28*).

In summary, this survey showed that 10 of the 19 surface mutants defined a majority class, exhibiting Bus, Bah, and Verde2-resistant/Verde1-sensitive phenotypes. Seven others exhibited weaker phenotypes and one (*bus-13*) was anomalous in being killed by both Verde2 and Verde1, despite its resistance to *M. nematophilum*.

### Molecular identification

Most of the genes discussed in the following sections were identified by whole genome sequencing (Sarin *et al*. 2008) of strains carrying one or more surface mutants mutation, for which genetic map positions had been determined to ± 0.5 centiMorgan/1,500 kb. Candidates were thus readily identified, despite the sometimes large background of irrelevant sequence alterations. Identification was confirmed by transgenic rescue or sequencing of additional alleles. These strategies worked effectively for the 10 genes discussed below; however, they yielded no good candidates for the previously reported genes *bus-3*, *bus-14*, *bus-15*, and *bus-16* (Gravato-Nobre *et al*. 2005). Molecular identities and biochemical similarities are summarized in Table 3. In the following sections, the 10 surface-modifying genes newly identified in this work are discussed in more detail.

**Table 3.** Molecular identities.

Gene name	Cosmid name	Biochemical features	Comment/reference
*srf-2*	F59C6.8	Glycosyl transferase GT92	This paper
*srf-3*	M02B1.1	UDP sugar transporter	Ref. 1
*srf-5*	F54B11.10	Small secreted protein	This paper
*bus-1*	R03H4.6	Acyl transferase	Ref. 3
*bus-2*	K08D12.5	Glycosyl transferase	Ref. 5
*bus-4*	T22B11.2	Glycosyl transferase	Ref. 5
*bus-5*	F53B1.4	TDP sugar dehydratase	This paper
*bus-6*	F52E1.9	PIP phosphatase	This paper
*bus-8*	T23F2.1	ALG2 mannosyltransferase	Ref. 4
*bus-10*	ZK596.3	Membrane protein	This paper
*bus-12*	JC8.12	UDP sugar transporter	Ref. 5
*bus-13*	T07H8.6	PTR receptor family	*ptr-15;* this paper
*bus-17*	ZK678.8	Glycosyl transferase	Ref. 2
*bus-18*	F55A11.5	Acyl transferase	*acl-10;* Ref. 3
*bus-19*	T07F10.4	Transporter TMEM41A	Ref. 2
*bus-21*	F56D1.5	Steroid dehydrogenase	*dhs-5;* this paper
*bus-22*	F37A4.3	Glycosyl transferase	This paper
*bus-24*	Y11D7A.9	GPI attachment factor	This paper
*bus-28*	M03F8.1	Membrane protein	This paper

References: Ref. 1, Höflich *et al*. (2004); Ref. 2, Yook and Hodgkin (2007); Ref. 3, Gravato-Nobre and Hodgkin (2008); Ref. 4, Partridge *et al*. (2008); Ref. 5, Gravato-Nobre *et al*. (2011).

### srf-2

**Fig. 2.**
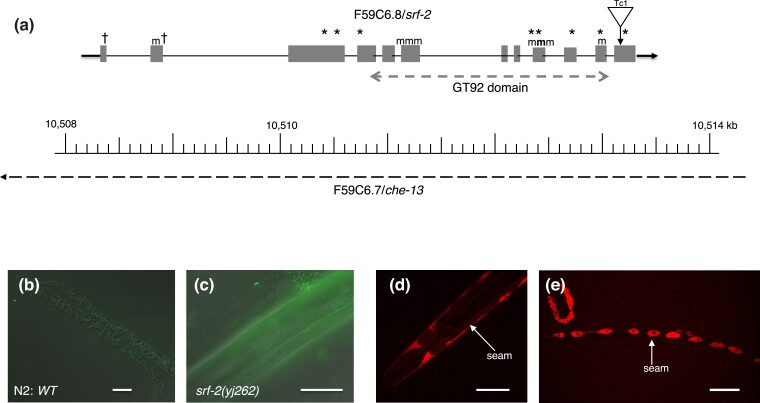
Structure and expression of *srf-2.* a) Genomic organization and mutations of *srf-2* on Linkage Group I. The gene is nested within *che-3* (F59C6.7), which is transcribed on the opposite strand. Sites of missense (m), nonsense (*), splice site (†) mutations, and Tc1 insertion are indicated; details are provided in Supplementary Table 2. The extent of GT92 domain is shown. b) and c) Fluorescent WGA lectin staining of N2 wild type (b) and *srf-2* (c). d) and e) *srf-2* promoter expression in adult d) and larvae e): strong expression in seam cells. Scale bars b)–e) ca. 50 microns.

Previous work led to the isolation of several alleles of *srf-2* on the basis of altered surface antigenicity (Politz *et al*. 1990; Link *et al*. 1992). Screens or selections for resistance to infection by *M. nematophilum* and *Leucobacter musarum* Verde2 generated many more, all of which had similar Bus and Bah phenotypes, and increased surface staining with fluorescent WGA lectin (Fig. 2, b and c). Detailed genetic mapping narrowed the location of the gene to a 100-kb region on Linkage Group I. Whole genome sequencing of a strain carrying the reference allele *yj262* suggested that *srf-2* = F59C6.8, a predicted protein-coding gene within the candidate region. The identification was confirmed by phenotypic rescue using transgenic constructs and by sequencing multiple alleles of the gene (Fig. 2; Supplementary Table 2). The original allele *yj262* proved to be a missense alteration (Ser346Arg) but the other identified mutations included a donor splice site mutation, an opal nonsense mutation, and a Tc1 transposon insertion, all of which are likely to be null alleles.

F59C6.8 (*srf-2*) encodes a 515 aa protein, which includes a predicted glycosyltransferase domain belonging to the GT92 family (a subset of DUF23). This ∼250 aa domain is found in at least 60 other *C. elegans* genes, including 2 previously characterized genes, *bah-1* (Drace *et al*. 2009) and *galt-1* (Titz *et al*. 2009). Mutations of the latter gene were isolated as a result of their resistance to a mushroom galectin toxin, and *galt-1* was shown to encode a manganese-dependent galactosyltransferase activity, which is probably required for toxin binding to an intestinal galectin receptor (Butschi *et al*. 2010). The GT92 family is phylogenetically widespread, being present in the genomes of many bacteria, plants, invertebrates, and fish (Hansen *et al*. 2012). However, it appears to be absent from mammals and consequently has been little studied as yet. Three genes containing GT92 domains in *Arabidopsis* have been examined in detail and all were found to encode functional beta-1,4 galactan synthases (Ebert *et al*. 2018), so galactosyltransferase activity may be the general function of this domain. The nematode protein BAH-1 has yet to be analyzed biochemically. Two other *bah* genes (*bah-2* and *bah-4*) also encode GT92 proteins (our unpublished results: O’Rourke *et al.*, in preparation). Null mutations of these 5 *C. elegans* genes are all viable, whereas a sixth GT92 gene, *subs-4* = Y47D3B.1, has been found to be essential and required for surface integrity as well as affecting susceptibility to pathogens (O’Rourke *et al.* in preparation).

Most of the identified missense mutations of *srf-2* affect the GT92 domain, the extent of which is indicated by the double-headed arrow in Fig. 2a and boldface in Supplementary Table 2. Sequence changes are listed in Supplementary Table 2; the majority of these affect residues that are conserved among homologous or paralogous GT92 genes. More detailed biochemical interpretation is difficult in the absence of much structural or enzymatic information about the GT92 domain.

The expression pattern for *srf-2* was explored using a promoter construct driving dsRedII; transgenic animals carrying this construct exhibited strong fluorescence in the seam cells (Fig. 2, c and d).

### srf-5

Mutations of *srf-5* were originally isolated on the basis of altered surface antigenicity, like those affecting *srf-2* (Link *et al*. 1992). Most of these, other than the reference allele *ct115*, are no longer available for investigation, but an additional allele (*e3147*) was isolated from selections for survival on *Leucobacter* Verde2. Whole genome sequencing revealed that *srf-5(ct115)* strains carried a nonsense mutation (Trp32Opal) in F54B11.10. Two further mutations in this gene were identified by sequencing (*e3147 =* Cys70Tyr) or from the Million Mutation Project (*gk424525* = Cys59Tyr), both of which lead to missense alterations in conserved cysteines. Correct identification was confirmed by transgenic rescue. All 3 alleles failed to complement each other and exhibited identical phenotypes with respect to sensitivity to pathogens. Since *ct115* is a nonsense mutation, it is likely that these are all null alleles.

**Fig. 3.**
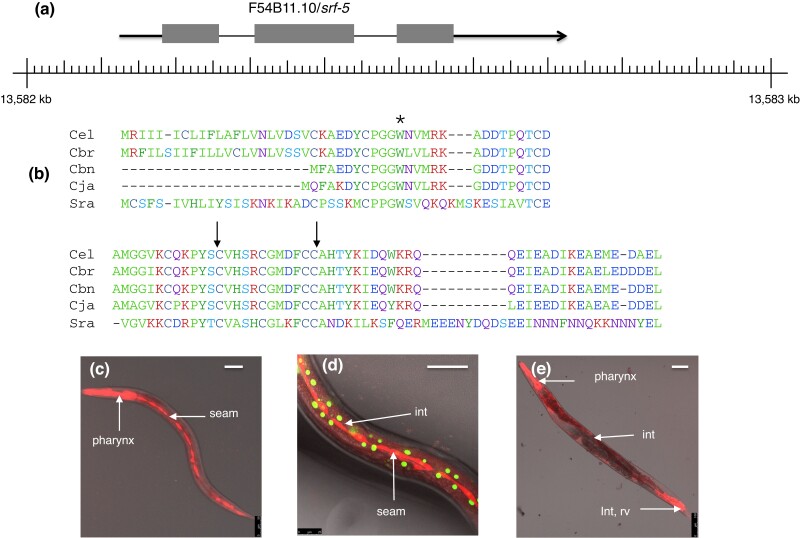
Structure and expression of *srf-5.* a) Genomic organization and mutations of *srf-5* on Linkage Group X. b) Protein sequence alignments for SRF-5 and orthologs in *Caenorhabditis* spp. (*elegans*, *briggsae*, *brenneri*, *japonica*) and *Strongyloides ratti.* Color code: red, positive; blue, negative; light blue, hydroxyl; purple, polar; green, hydrophobic; dark green, aromatic. c), d), and e) *srf-5* bicistronic rescuing construct (strain CB7039) revealing expression in seam cells, pharynx, intestine, and rectal cells. Hypodermal nuclei are marked with GFP in panel d). Scale bars ca. 50 microns.

F54B11.10 (*srf-5*) is predicted to encode a small (99 aa) cysteine-rich protein with a probable N-terminal signal sequence (Fig. 3b). The small size of the gene may explain why few mutations have been found for *srf-5*. Comparable small proteins with strong homology to *srf-5* can be found in most sequenced *Caenorhabditis* genomes (ranging in size from 78 aa in *C. brenneri* to 102 aa in *C. angaria*), as well as in more distantly related nematodes (117 aa in *Panagrellus redivivus* and 115 aa in *Stronglyloides ratti*), so this gene appears to be well conserved. Selected protein alignments are shown in Fig. 3b.

The *srf-5(ct115)* mutant was rescued using a bicistronic construct (Gravato-Nobre *et al*. 2011) in order to examine expression patterns. Efficient rescue of pathogen sensitivity was observed using unusually low concentrations of this construct, while higher concentrations resulted in embryonic lethality. Strong expression was observed in seam cells, as well as weaker expression in the intestine, rectal valve, and pharynx (Fig. 3, c–e).

The nature of the predicted SRF-5 protein, and the lack of any obvious enzymatic motifs in its sequence, suggested that it might be an integral component of the cuticle or surface coat, in contrast to the other *srf* and *bus* genes, which mostly encode proteins implicated in glycosylation or other posttranslational modifications (Table 3). In order to examine SRF-5 protein distribution in vivo, various constructs encoding C-terminal fusions between SRF-5 and fluorescent reporters were prepared, but all of these exhibited high levels of embryonic toxicity when injected into worms, even at very low concentrations. An attempt was made to circumvent this problem by using transgenes encoding an amber mutant of *srf-5* (W32Amber), which would potentially enable tagging the protein with an unnatural amino acid (Davis and Greiss 2018), but these transgenes unexpectedly rescued the Srf mutant phenotype when crossed into *srf-5* worms. This effect was presumably a consequence of multicopy arrays permitting a low level of translational readthrough of the amber codon. This observation suggests that SRF-5 is required only in small amounts, which would not be consistent with a role as a major structural component. Quantitative analyses (Cao *et al*. 2017) indicate that this gene is expressed at very low levels.

### bus-5

Mutations of *bus-5* were frequently isolated in selections for resistance to infection by *M. nematophilum* (Gravato-Nobre *et al*. 2005) or *Leucobacter* Verde2 (Supplementary Table 1). An additional Bah allele was obtained by Creg Darby in screens for resistance to Yersinia biofilm formation (Darby *et al*. 2007). All 20 of these mutants were viable on lawns containing either *M. nematophilum* or *Leucobacter* Verde2, and all were inviable on lawns containing *Leucobacter* Verde1. However, they exhibited a striking range of different sensitivities to bleach or detergent and to *Yersinia* biofilm formation (Supplementary Table 3). Some alleles were similar to the wild type with respect to these phenotypes, whereas only a few alleles exhibited a complete larval Bah phenotype, and these alleles showed the strongest sensitivity to bleach and to detergent. Weaker alleles such as the class F allele *e2985* exhibited a weak Dar phenotype when growing on Verde2 lawns, indicating incomplete resistance. On the basis of these phenotypes, isolates could be placed in an allelic series of 6 classes, of which the most severe (class A) exhibited a complete Bah phenotype and strong sensitivity to bleach or detergent. One class A allele, *bus-5(br19)* (missense Gly142Glu), has been found to be an optimal strain for drug and toxin assessment (Xiong *et al*. 2017), as a consequence of its substantially higher sensitivity to many drugs.

The *bus-5* gene was identified by genetic mapping followed by whole genome sequencing of a strain carrying a weak (class F) allele, *e2985*, which suggested that *bus-5* = F53B1.4. This identification was confirmed by finding sequence alterations in this gene for 18 other alleles of *bus-5* and by transgenic rescue. The gene encodes a predicted protein of 342 aa, with significant similarity to dTDP-D-glucose 4,6-dehydratase (TGDS) from a variety of organisms.

**Fig. 4.**
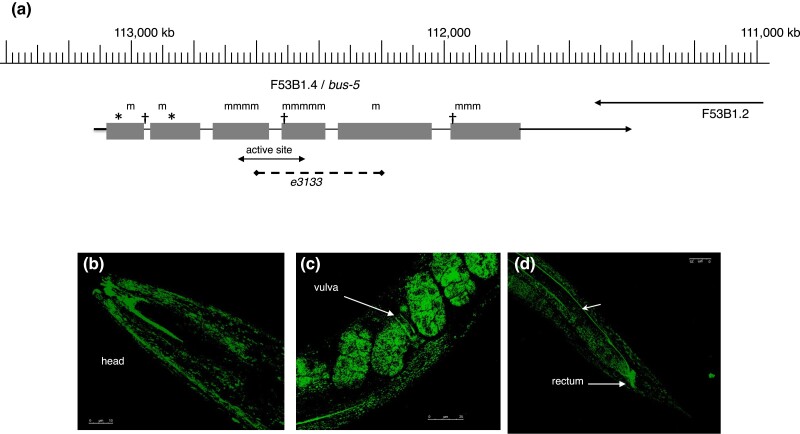
Structure and expression of *bus-5.* a) Genomic organization and mutations of *bus-5* on Linkage Group X. Sites of missense (m), nonsense (*), splice site (†) mutations are indicated, along with the extent of the putative active site and the region deleted by *e3133* b), c), and d). Confocal fluorescence images of adult worms expressing a rescuing bicistronic *bus-5* reporter (strain CB7418), showing hypodermal and rectal expression. In c), note vulval and embryonic expression; in d), note possible expression in the intestinal basement membrane (short arrow). Scale bars 10 microns b), 25 microns c) and d).

Research cited below suggested that this gene might be essential, in contrast to the viability observed for the strongest alleles reported here (including 2 nonsense mutations). We, therefore, used CRISPR/Cas9 to generate a 455-bp deletion allele, *e3133*, which removes most of the exons 3–5, including the predicted active site (Fig. 4, Supplementary Table 3, Supplementary Methods). Animals homozygous for this allele were fully viable and exhibited phenotypes identical to those of 5 other class A alleles, which we, therefore, concluded are all null alleles. In contrast, none of 3 different splice junction mutations resulted in a null phenotype.

The protein encoded by F53B1.4 has also been studied in vitro, as part of a study of nematode rhamnose biosynthesis (Feng *et al*. 2016). By expressing this protein in *E. coli*, these authors identified it as RML-2, catalyzing the second step in a biosynthetic pathway for rhamnose. Other steps in the pathway were encoded by *rml-1/*K08E3.5, *rml-3*/C14F11.6, *rml-4/*C01F1.3, and *rml-5/*Y71G12B.6. Most of these activities appeared to be essential for viability, on the basis of RNAi knockdown experiments, which suggested that rhamnose biosynthesis is an essential function for nematodes. However, our null mutants of *bus-5/rml-2* were clearly viable, implying that this step in rhamnose biosynthesis is dispensable or else is not uniquely provided by *bus-5/rml-2*. Nevertheless, the sequence features of BUS-5 strongly implicate it in some kind of carbohydrate biosynthetic pathway.

Defects in human TGDS lead to Catel–Manzke syndrome, which is characterized by a unique form of hyperphalangy (Ehmke *et al*. 2014; Pferdehirt *et al*. 2015). The biochemical function of human TGDS is not known. It cannot be acting in rhamnose biosynthesis, because this sugar is not found in humans (Wagstaff *et al*. 2021), but TGDS is presumed to be involved in proteoglycan biology. Catel–Manzke syndrome occurs most commonly as a result of a missense mutation (Ala100Ser). The corresponding residue of BUS-5 is Ala92, which is mutated to threonine in a class D allele of *bus-5*, *e3129*. We, therefore, constructed a mutant of *bus-5* encoding Ala92Ser but found that a transgene expressing this version was able to rescue the class A mutant *bus-5(e2801).* This observation suggests that *bus-5* does not provide a directly useful model for the human syndrome, but nevertheless it may provide insights.

The expression pattern for *bus-5* was examined using a rescuing bicistronic construct containing *bus-5* and GFP; fluorescence was observed in hypodermal and rectal tissues and possibly in the intestinal basal lamina (Fig. 4, b–d).

### bus-6

**Fig. 5.**
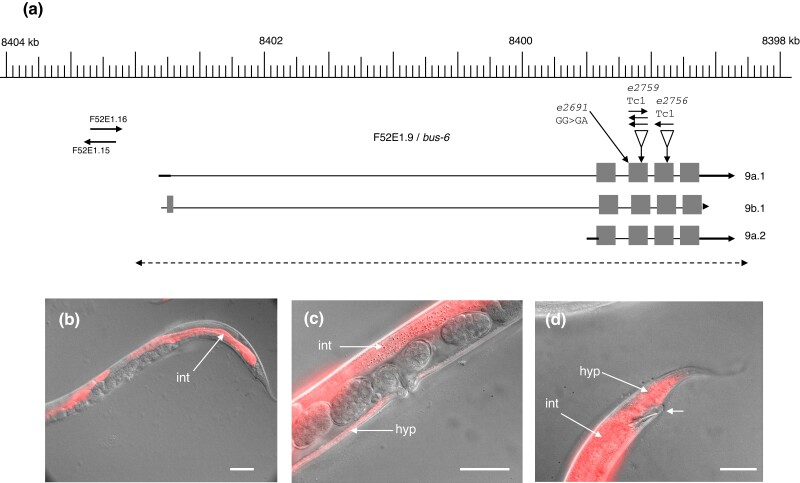
Structure and expression of *bus-6.* a) Genomic organization and mutations of *bus-6* on LGV. Sites of mutation and Tc1 insertion are indicated. The dashed line marks the extent of the 4-kb fragment used to examine expression. b)–d) Merged DIC and fluorescence images of *M. nematophilum*-infected *bus-6* worms expressing a bicistronic *bus-6* reporter (strain CB6923), showing expression in the intestine (int) and hypodermis (hyp). The short arrow in panel d) indicates a weak Dar response, symptomatic of incomplete rescue by this reporter. Scale bars b)–d) ca. 50 microns.

Mutations of *bus-6* were recovered from initial *bus* screens (Gravato-Nobre *et al*. 2005) using both EMS and the mutator *mut-7*; these mutants were viable on Verde2 lawns but exhibited a Dar phenotype, consistent with incomplete resistance. Similarly, they exhibited an incomplete Bah phenotype on *Yersinia* lawns, with some biofilm attachment to larvae. They were also viable on Verde1, in contrast to mutants of *srf-2*, *srf-5*, and many *bus* genes (Table 1). The gene was identified as F52E1.9 on the basis of genetic mapping and whole genome sequencing of strains carrying the reference allele *e2691*. Four independent mut-7-induced alleles were found to carry Tc1 insertions in exons of this gene, and 2 independent EMS-induced alleles, including *e2691*, carried the same splice acceptor mutation (Fig. 5a). Another *mut-7* allele, *e2728*, is probably a deletion or rearrangement of F52E1.9, because it could not be PCR amplified with standard primers for this locus.

The gene structure on WormBase for F52E1.9/*bus-6* suggests that it has 3 isoforms (Fig. 5a), encoding proteins of 169 aa (F52E1.9a.1,2) and 181 aa (F52E1.9b.1). These proteins contain a phosphatidylinositol 4,5-bisphosphate 4-phosphatase domain, which is also found in the paralogous gene Y71H2AM.2. This encodes the apparent 251 aa nematode ortholog of human PIP4P1, but no biological function has yet been ascribed to this *C. elegans* gene (Davis *et al*. 2022). Both *bus-6* and Y71H2AM.2 are conserved in other *Caenorhabditis* species and at least some other nematode genera (Davis *et al*. 2022).

A bicistronic reporter, including the whole coding region of all isoforms linked to red fluorescent protein T (RFPT), was constructed in order to examine expression. Transgenic animals carrying this reporter exhibited incomplete rescue of the Bus-6 phenotype (Fig. 5d), suggesting that additional sequence may be required for full expression, such as the upstream noncoding RNAs (Fig. 5a). Transgenic animals exhibited strong fluorescence in the intestine and variable weaker fluorescence in hypodermal tissues (Fig. 5, b–d).

The relatively weak mutant phenotypes exhibited by *bus-6* mutants and the gene product's possible involvement in phosphatidylinositol signaling suggest that *bus-6* may only have modulatory effects on surface properties. Alternatively, it may be partly redundant with Y71H2AM.2.

### bus-10 and bus-28

Mutations of *bus-10* were recovered from initial *bus* screens for *M. nematophilum* resistance (Gravato-Nobre *et al*. 2005) using both EMS and *mut-7*, a mutator strain with increased transposon mobilization (Ketting *et al*. 1999). Additionally, these mutants were found to be viable on Verde2 lawns but exhibited a weak Dar phenotype; they were inviable on Verde1 lawns (Table 1).

**Fig. 6.**
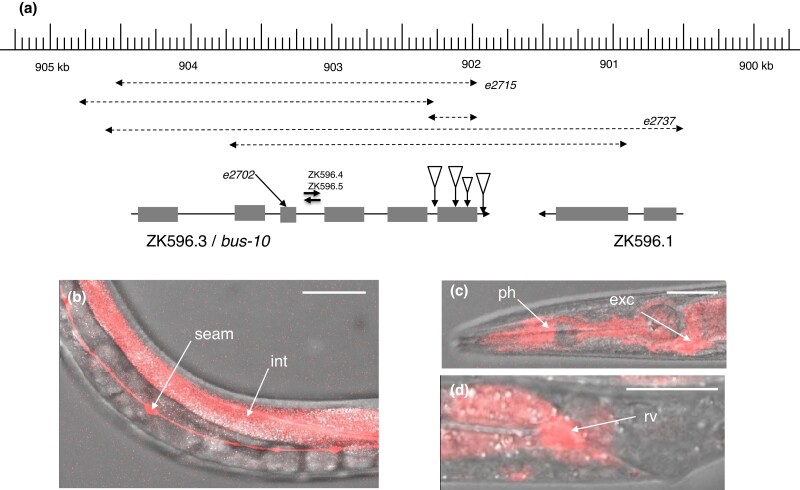
Structure and expression of *bus-10*. a) Genomic organization and mutations of *bus-10* on LGIV. Sites of mutation, deletion (dashed lines), and transposon insertion are indicated (large triangles Tc1, small triangle Tc4). b)–d) Merged DIC and fluorescence images of worms expressing a rescuing bicistronic *bus-10* reporter (strain CB6957), showing expression in intestine (int), seam cells (seam), pharynx (ph), excretory cell (exc), and rectal valve (rv). Scale bars b)–d) ca. 50 microns.

The gene was identified as ZK596.3 by genetic mapping and whole genome sequencing of a strain carrying the EMS-induced reference allele *e2702*. Sequencing this locus in the mutator-induced alleles revealed that it is a hotspot for transposon insertion and deletion. Eight of the *mut-7* alleles were found to be insertions of transposon Tc1 or Tc4, and 5 were deletions centered on exon 6, the probable location of the hotspot (Fig. 6a). Some of the deletions covered most of the gene and in some cases the adjoining gene, *srlf-30* (ZK596.1), as well as deleting 2 internal genes for noncoding RNAs, ZK596.4 and ZK596.5. None of these deletion alleles exhibited any detectable phenotypic differences from the *bus-10* reference allele *e2702*, which is a nonsense mutation (Trp119Opal) so *srlf-30*, ZK596.4, and ZK596.5 appear to be nonessential genes.

**Fig. 7.**
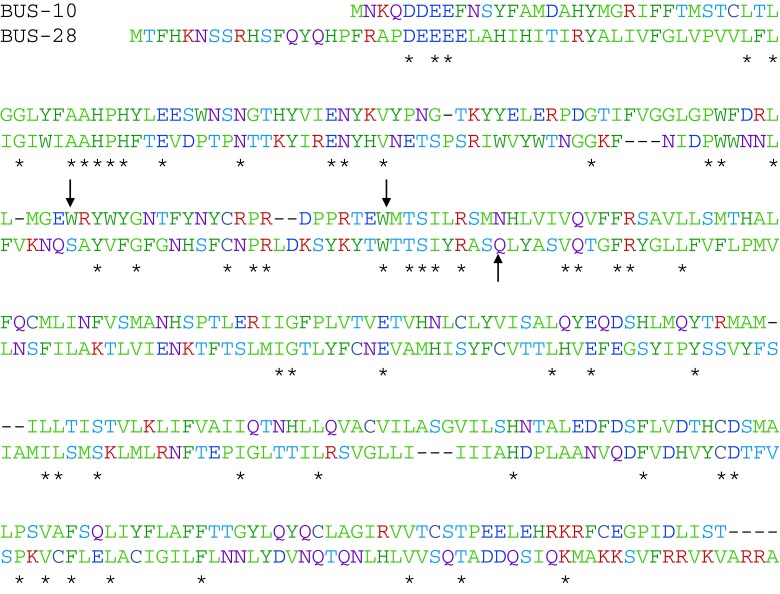
Sequence alignment of BUS-10 and BUS-28. Color code as in Fig. 3. Arrows indicate the location of nonsense mutations in *bus-10* and *bus-28*. Asterisks mark identical residues.

The *bus-10* gene encodes a predicted 322 aa protein with high hydrophobicity (Fig. 7), which was, therefore, inferred to be an integral membrane protein. It has no obvious homology to other proteins outside of the genus *Caenorhabditis*, so its biochemical function cannot be predicted at present. We examined the *bus-10* expression pattern using a bicistronic construct with *bus-10* sequences linked to TagRFP-T; this construct was able to rescue the Bus-10 phenotypes in *bus-10* transgenic worms and exhibited expression in many tissues, most notably the seam cells, pharynx, intestine, rectal cells, and excretory gland (Fig. 6, b–d).

Searching for *bus-10* paralogs revealed that M03F8.1 is the only other *C. elegans* gene with significant similarity to *bus-10* (score 5e−14), encoding a protein of similar size (344 aa) and hydrophobicity (Fig. 7). A nonsense mutation of this gene (*gk236264* = Q146amber) has been generated by the Million Mutation Project (Thompson *et al*. 2013) and is present in the multiply mutant strain VC20170, which was found to have a Bus phenotype. The nonsense mutation was, therefore, extensively crossed onto a wild-type (N2) background, and the resulting strain still exhibited a Bus phenotype as well as being partly resistant to Verde2 and hypersensitive to Verde1 (Table 1). M03F8.1 has, therefore, been assigned a *bus* gene name, *bus-28*.

Both *bus-10* and *bus-28* appear to be well conserved within the *Caenorhabditis* genus, although no obvious homologs have been found in other taxa. A double mutant strain, [CB7516 = *bus-10(e2702); bus-28(gk236264)*] was constructed and found to be indistinguishable from *bus-10(e2702)* alone, so these 2 genes do not appear to be redundant, despite their similarity. Notably, RNAi knockdown tests on both *bus-10* and *bus-28* failed to phenocopy their null mutant phenotypes, suggesting that only low-level expression is required for their function. High-throughput expression surveys summarized on WormBase (Davis *et al*. 2022) indicate that *bus-28* expression is predominantly hypodermal.

### bus-13/ptr-15

The single *bus-13* mutation, *e2710*, exhibited an unusual phenotype with respect to pathogen sensitivity (Table 1). Whole genome sequencing suggested that *e2710* corresponded to a missense mutation (Glu364Lys) in T07H8.6, the Patched-related gene *ptr-15* (Zugasti *et al*. 2005). The identity of *bus-13* as *ptr-15* was confirmed by establishing transgenic lines carrying a 5-kb construct including the coding and upstream sequences of T07H8.6. These transgenes fully rescued all the mutant phenotypes of *bus-13(e2710).* Further investigation revealed that *ptr-15/bus-13* is an essential gene with complex and unique properties, which will be reported elsewhere (Kuwabara *et al.*, in preparation).

### bus-21/dhs-5

A mutation of *bus-21*, *e2992*, was recovered from the initial selections for *M. nematophilum* resistance, and slightly stronger alleles (*e2997* and *e2998*) were obtained by means of a non-complementation screen. These mutants exhibited a strong Bus phenotype on lawns containing *M. nematophilum* and were fully viable on Verde2 mixed lawns and fully inviable on Verde1 mixed lawns. They were also noticeably skiddy in movement and significantly bleach sensitive (Table 2), as well as occasionally exhibiting molting defects and rod-like larval lethality (3.3%).

Detailed genetic mapping suggested that *bus-21* might correspond to a named gene, F56D1.5 = *dhs-5*, but RNAi knockdown tests on this gene failed to phenocopy the Bus-21 phenotypes. However, sequencing of *dhs-5* in *bus-21* mutants revealed that *e2992* and *e2997* both carry missense mutations in conserved residues of *dhs-5*: Cys239Tyr for *e2992* and Gly120Glu for *e2997*.

F56D1.5 encodes a 378 aa protein with clear homologs in many other nematode species (*Brugia malayi*, *Onchocerca volvulus*, *Strongyloides ratti*, etc.). It has 38 paralogs in the *C. elegans* genome, most of which have been assigned to the gene classes *dhs* (DeHydrogenase, Short chain) or *stdh* (STeroid DeHydrogenase). F56D1.5 has previously been assigned the gene name *dhs-5* but in fact it is most similar in sequence to a group of 7 genes (TreeFam set TF314591) with homology to steroid dehydrogenases (*dhs-5*, *dhs-27*, *stdh-1* to *stdh-4*, and *let-767*). The last of these, *let-767*, has been examined in detail as a result of the isolation of 3 lethal alleles (Kuervers *et al*. 2003). These mutants exhibited hypersensitivity to dietary limitation of cholesterol, suggesting that the encoded protein acted on a sterol derivative. Mutants also exhibited defects in embryogenesis, oogenesis, and molting. Expression analysis indicated that *let-767* was expressed primarily in the intestine, although the properties of a weak maternal-effect lethal allele indicated that its product could also be provided to embryos via oogenesis. Subsequent in vitro investigation of LET-767 (Entchev *et al*. 2008) revealed that it is a major 3-ketoacyl-CoA reductase required for the bulk production of worm ceramides. Whether DHS-5/BUS-21 has similar biochemical functions remains to be seen; the skiddiness and bleach sensitivity of *dhs-5* mutants suggest that it is essential for normal surface function, in addition to affecting pathogen susceptibility. High-throughput surveys summarized on WormBase (Davis *et al*. 2022) suggest that is expressed in hypodermis as well as vulval and enteric muscles.

### bus-22

**Fig. 8.**
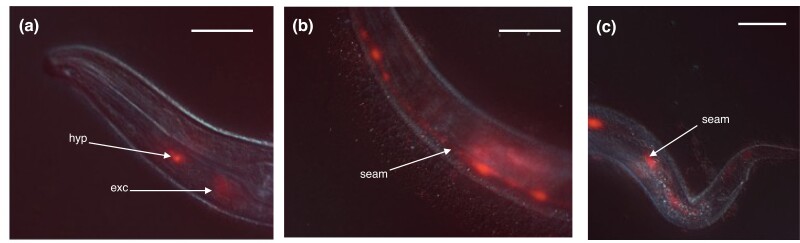
Expression of *bus-22.* Merged DIC and fluorescence images of head a), body b), and tail c) of worms expressing a rescuing bicistronic *bus-22* reporter transgene (strain CB7439). Expression sites in a head hypodermal cell (hyp), excretory duct cell (exc), and seam cells (seam) are indicated. Scale bars ca. 50 microns.

Mutants of *bus-22* were recovered from selections for viability on Verde2 lawns and found to exhibit a typical phenotype of resistance to *M. nematophilum*, *Yersinia pseudotuberculosis*, and *Leucobacter* Verde2, and hypersensitivity to *Leucobacter* Verde1 (Table 1). The molecular identity of *bus-22* was determined by genetic mapping followed by whole genome sequencing of a strain carrying the reference allele *bus-22(e2798*), which was found to contain a nonsense mutation, Arg16Opal, in F37A4.3. Further sequencing of a different allele, *e3108*, revealed that this carried a different nonsense mutation, Trp182Amber, in the same gene. Consistent with this observation, the mutant phenotypes of *bus-22(e3108)* were suppressed by the amber suppressor mutation *sup-7(st5*). The identification of *bus-22* was confirmed by the transgenic rescue of *bus-22(e2798)* by bicistronic constructs of F37A4.3 and TagRFP-T (see Supplementary Materials). These constructs caused significant toxicity at a standard injection concentration of 30 ng/μl and viable lines were only obtained using a much lower concentration, 1 ng/μl. Nevertheless, these lines exhibited full rescue of *bus-22* phenotypes, being fully sensitive to *M. nematophilum* and Verde2 and resistant to Verde1. Expression of the RFP marker was detectable in seam cells, the excretory duct cell, and head hypodermal cells (Fig. 8).

Two million mutation project (MMP) strains carrying conserved missense mutations of F37A4.3 were examined for resistance phenotypes. One of these, carrying *gk850019* (Pro75Leu), exhibited a weak Bus phenotype, with some survival on Verde2 lawns but much less than the nonsense mutants of this gene. Heterozygous animals, of genotype *gk850019/e3108*, were also tested and found to resemble *gk850019* homozygotes. The other MMP strain, carrying *gk605625* (Pro220Ser), appeared to be wild type in phenotype.

Close orthologs of *bus-22* can be found in most *Caenorhabditis* genomes. Genome and proteome annotation of BUS-22, the predicted protein encoded by F37A4.3 (272 aa with a signal sequence), suggested a possible similarity to the previously characterized BUS-4 (Gravato-Nobre *et al*. 2011; Parsons *et al*. 2014). Sequence analysis in PANTHER (Thomas *et al*. 2003) indicates that there are 10 paralogs of *bus-4*, most of which encode proteins of similar size and are well conserved among different *Caenorhabditis* species. BUS-22 appears to be the most diverged of this set of predicted glycosyltransferases, most of which contain a Fringe-like domain, though this is not recognizable in BUS-22. *Drosophila* Fringe, the founding member of this protein class, acts as a fucose-specific beta 1,3 *N*-acetylglucosaminyltransferase modulator of Notch receptor proteins, acting to elongate *O*-linked fucose residues (Moloney *et al*. 2000). This receptor modification appears to be essential in most animals. Substantial alterations in *O*-linked glycans were observed in analyses of *bus-4* mutants (Parsons *et al*. 2014), indicating a similar role in *C. elegans*. Whether *bus-22* mutants exhibit similar changes has not been determined. A double mutant, CB7566 = *bus-22(e2798); bus-4(br4)*, was constructed in order to test for possible redundancy and found to exhibit identical phenotypes to *bus-4(br4*) alone.

### bus-24

A mutation of *bus-24*, *e3020*, was obtained from selections for resistance to Verde2. Three additional alleles were recovered from selections for resistance to Verde1, using *bus-10* and *srf-5* strains which are hypersensitive to Verde1 (Loer *et al*. 2015). All 4 alleles exhibited significant resistance to both pathogens, like mutants of some of the other *bus* genes (Table 1: *bus-1*, *bus-6*, and *bus-18*), with *e3020* exhibiting slightly stronger mutant phenotypes than the other alleles.

Genetic mapping and whole genome sequencing indicated that *bus-24* corresponds to Y11D7A.9: both *e3020* and *e3034* cause missense changes (Ser88Leu, Gly120Glu) in conserved residues of the protein encoded by Y11D7A.9. The 297 aa predicted protein belongs to a *C. elegans* set of 8 paralogous proteins of similar size, all of which have similarity to the phylogenetically conserved protein PGAP2. This protein is required in the Golgi apparatus for remodeling the glycosylphosphatidylinositol anchor in order to permit the C-terminal glypiation of selected proteins, which are thereby anchored to the external leaflet of the cell membrane. In the paralogous set of *C. elegans* genes, one (T04A8.12) appears to be orthologous to PGAP2 and it has, therefore, been assigned the gene name *pgap-2*. A deletion mutant of this gene is sterile (Ihara *et al*. 2017). BUS-24 and other members of the set are substantially less similar to mammalian PGAP2 than *C. elegans* PGAP-2, so their functions cannot yet be reliably inferred, but it is reasonable to speculate that they are involved in some kind of posttranslational modification of proteins, like PGAP2. Seven of these genes, including *pgap-2* and *bus-24*, are well conserved in other nematode genomes.

High-throughput expression analyses reported on WormBase (Davis *et al*. 2022) indicate that *bus-24* is expressed in the embryonic and larval hypodermis and possibly in germline precursor cells.

## Discussion

The survey and analyses reported here substantially expand the set of genes known to be required for the production of a normal external surface in *C. elegans*. The fact that the 10 genes described here were identified on the basis of a mutant phenotype of resistance to one or more bacterial pathogens demonstrates that they all have significant biological roles.

These genes, as well as the 9 *srf* and *bus* genes previously identified at a molecular level, have been defined on the basis of mutant resistance to bacterial pathogens, but some of the *srf* and *bus* mutants have been shown to have a contrasting phenotype of *decreased* resistance to fungal pathogens that attack by surface attachment, such as *Duddingtonia flagrans* and *Drechmeria coniospora* (de Gives *et al*. 1999; Rouger *et al*. 2014). The chemical composition of the worm's surface probably reflects a complex trade-off between susceptibility to the many different microbial pathogens encountered by *C. elegans* in its natural environments. However, no significant natural polymorphisms have yet been reported for these genes.

As well as affecting susceptibility to pathogens, some of these genes demonstrably have important additional functions, summarized in Table 2. Many affect the permeability of the cuticle, as assayed most rapidly by means of bleach sensitivity tests. Mutant bleach sensitivity is strongly correlated with sensitivity to detergents such as SDS and with increased susceptibility to many drugs (Xiong *et al*. 2017). Most mutants with conspicuous cuticle permeability defects also displayed defective “skiddy” movement (Skd) when moving on a solid agar surface, which presumably reflects inadequate traction between the body of the worm and the underlying agar surface. The poor crawling mobility of these mutants is not due to neuromuscular defects, because they exhibit thrashing rates similar to the wild type, when swimming freely in liquid (Supplementary Table 4). The *bus-19* and *bus-21/dhs-5* mutants exhibited reduced thrashing rates, but not enough to explain their poor crawling mobility.

In addition to altering attachment by bacteria and fungi, we have shown previously that many *bus* genes can affect intraspecies recognition, because during mating wild-type males spend less time in contact with mutant hermaphrodites than with wild-type hermaphrodites (Gravato-Nobre *et al*. 2011). None of the 10 mutants discussed in the present paper has been examined using the mating contact assay. All can be mated successfully with wild-type males, but it is probable that at least some have similar recognition defects to those previously reported for *srf-3*, *bus-2*, and other *bus* mutants. Interspecies recognition is also likely to be affected, such as is seen in the predatory attacks of *Pristionchus pacificus* on *C. elegans* (Quach and Chalasani 2020).

The 19 genes discussed in this report are conserved among all well-sequenced *Caenorhabditis* spp. and most have identifiable orthologs in more distantly related nematodes. Several belong to multigene families, such as the GT92 set, which raises the possibility of redundancy, but none of the double mutants so far constructed have exhibited any unexpected phenotypes (Gravato-Nobre *et al*. 2011, this paper), which argues against redundancy. None of the redundant genes defined by Tischler *et al*. (2006) appear to be involved in surface biology.

Most of these genes have nonlethal null phenotypes. Exceptions are *bus-8* and *bus-13/ptr-15*, although the inviability of *bus-8* null embryos appears to be due to failures in embryonic cell migrations and ventral enclosure, rather than in defective surface integrity (Partridge *et al*. 2008). The lethal null phenotype of *bus-13* will be reported elsewhere (Kuwabara *et al.*, in preparation). In this context, the gene *glf-1* is also relevant: this gene encodes a homolog of UDP-galactopyranose mutase, which is required for the synthesis of galactofuranose (Novelli *et al*. 2009). Deletion mutants of *glf-1* display late embryonic or early larval lethal phenotypes indicative of defective surface coat synthesis (bleach and osmotic sensitivity, increased permeability, abnormal lectin staining, and skiddy movement). The lethality of these mutants precluded detailed pathogen testing. Nevertheless, the isolation of viable alleles of *bus-8* and *bus-13/ptr-15* demonstrates that screening for pathogen-resistant mutants can lead to the identification of essential genes required for surface coat synthesis.

Lethal effects were also observed for overexpression in some cases: for 2 of the 9 genes under discussion, *srf-5* and *bus-22*, the transgene constructs used to demonstrate mutant rescue and gene expression patterns caused conspicuous embryonic lethality when injected into wild-type worms. Dying transgenic embryos were recognized by the expression of the coinjection fluorescent markers. Transgenic lines could only be established by using thirty-fold lower concentrations of the wild-type *srf-5* and *bus-22* constructs, and several different translational fusion constructs of *srf-5* were found to be even more toxic, such that no transgenic lines could be established. These observations suggest that overexpression of SRF-5 or BUS-22 protein is toxic to the embryo, presumably by interfering with a vital process such as the formation of a functional ectodermal layer.

With the probable exception of *bus-1* (Gravato-Nobre and Hodgkin 2008), all 19 genes summarized in Tables 1 and 2 appear to affect most of the externally exposed surface of the worm, as assayed by adhesion to bacteria or by lectin staining. General surface alterations have also been observed for mutants of the 4 identified *bah* genes and for Verde1-resistant mutants; these classes will be discussed elsewhere.

A common feature of these genes (again with the exception of *bus-1*) is that many are strongly expressed in seam cells, as revealed by reporter gene constructs. Some, however, appear to be more strongly expressed in hypodermal tissues. Additional sites of expression were seen using some of the reporters, and yet more have been reported in the high-throughput expression analyses summarized on WormBase (Davis *et al*. 2022) but it is not clear whether these additional sites are valid or functionally significant.

Undoubtedly many more genes affecting nematode surface coat remain to be discovered, especially as RNAi seems to be relatively inefficient in knocking down the expression of surface-affecting genes. Are we only scratching the surface? Other mutants affecting surface properties include those defining additional *srf* genes (*srf-4*,*6*,*8*,*9*), but none of these affects *M. nematophilum* infection, as previously reported (Hodgkin *et al*. 2000). The molecular identities of the grossly pleiotropic genes *srf-4*, *srf-8* and *srf-9* (Link *et al*. 1992) remain unknown. Surprisingly, *srf-6* has recently been found to correspond to *nsy-1*, a neuronal gene encoding a p38 MAP kinase pathway component (Van Sciver *et al*. 2019), which suggests that neuronal signal transduction pathways can modulate the expression of surface antigens (Politz 2019). Similarly, chemotaxis mutants such as *tax-4* exhibit altered surface properties (Yook and Hodgkin 2007).

The molecular identity of these 10 genes provides some clues as to their biochemical functions. Three encode proteins implicated in glycosylation by virtue of sequence features: *srf-2*, *bus-5*, and *bus-22*, which makes them similar to the previously reported *srf-3*, *bus-2*, *bus-4*, *bus-8*, *bus-12*, and *bus-17*. Sequence similarities and glycan analyses implicate both *O*-linked glycosylation and *N*-linked glycosylation. However, the targets of such glycosylation remain uncertain; the significant end products might be glycolipids or complex glycans rather than glycoproteins. SRF-5 is one possible secreted target protein; conceivably this corresponds to the small surface protein detected by iodination (Blaxter 1993) although this protein was described as non-glycosylated. The similarity of BUS-24 to factors involved in glypiation raises the possibility that some of the surface components may be GPI-anchored to the glycolipid surface of the worm. However, BUS-24 is much less similar to mammalian PGAP2 than *C. elegans* PGAP-2.

Exactly how all the various mutants alter the surface at a biochemical level remains to be determined, and advances in this area will depend on a better molecular understanding of surface coat. Alterations in antigenicity, lectin binding, and glycan profiles have been demonstrated for many of the mutants (Politz *et al*. 1990; Link *et al*. 1992; Silverman *et al*. 1997; Cipollo *et al*. 2004; Gravato-Nobre *et al*. 2005; Palaima *et al*. 2010; Parsons *et al*. 2014), but the alterations are often only quantitative and may be indirect consequences of primary biochemical defects. Proteomic and glycomic investigations of nematode surface tissues are likely to be dominated by contributions from collagens and cuticlins, which are abundant proteins encoded by a large number of genes and subject to substantial posttranslational modification. The surface coat must usually constitute only a small percentage of the material extracted and examined in such surveys. Extraction using gentler methods has been explored and may provide a better route for biochemical analysis (Bada Juarez *et al*. 2019) but even this approach yielded complex mixtures of lipids and proteins.

Further genetic screens and suppressor selections, which are greatly facilitated by the various pathogens utilized in the present work, will lead to the identification of more factors involved in surface biosynthesis. The present work has revealed new genetic, biological, and biochemical aspects of this major element of nematode anatomy and biology.

## Supplementary Material

jkad056_Supplementary_Data

## Data Availability

Reference strains have been deposited with the Caenorhabditis Genetics Center. Supplemental material is available at G3 online.
