# Supplementary material for: Isolation and molecular identification of nematode surface mutants with resistance to bacterial pathogens
Source: G3 (Bethesda). 2023 Mar 13;13(5):jkad056. doi: 10.1093/g3journal/jkad056 (PMC10151413; doi:10.1093/g3journal/jkad056)
Supplement: jkad056_Supplementary_Data [file jkad056_supplementary_data.docx]

**Title:**

**SUPPLEMENTAL MATERIAL**

STRAIN LIST

SUPPLEMENTARY METHODS

SUPPLEMENTARY TABLE S1: Source of mutants

SUPPLEMENTARY TABLE S2: *srf-2* mutations

SUPPLEMENTARY TABLE S3: *bus-5* mutations

SUPPLEMENTARY TABLE S4: Thrashing rates

RELEVANT SEQUENCE DATA

SUPPLEMENTARY LITERATURE CITED

**Strain list**

AT6 *srf-2(yj262)*

AT10 *srf-3(yj10)*

CB5619 *srf-5(ct115)*

CB5609 *bus-1(e2678)*

CB5610 *bus-2(e2687)*

CB7549 *bus-4(br4)*

DC19  *bus-5(br19)*

CB5613 *bus-6(e2691)*

CB6177 *bus-8(e2883)*

CB5617 *bus-10(e2702)*

CB6667 *bus-12(e2977)*

CB5635 *ptr-15(e2710)*

CB7431 *bus-17(br2)*

CB6037 *bus-18(e2795)*

CB6338 *bus-19(e2912)*

CB6794 *dhs-5(e2997)*

CB6881 *bus-22(e2798)*

CB6978 *bus-24(e3020)*

CB7492 *bus-28(gk236624)*

CB7516 *bus-10(e2702) IV; bus-28(gk236264) V*

CB7566 *bus-22(e2798) III; bus-4(br4) IV*

Expression reporter strains:

CB6973 *unc-119(ed3); eEx711 [srf-2p::dsRed2; unc-119(+)]*

3.6 kb *srf-2* promoter construct (I: 10,505,472 – 10,509,100)

CB7039 *srf-5(ct115); eEx740[srf-5(W32amber)::gpd-2/gpd-3::Tag-RFP-T;*

*sur-5p::GFP]*

Rescuing bicistronic construct using 1.6 kb genomic *srf-5* (X: 13,581,746 - 13,583,339), amber mutation included to reduce toxicity.

CB7418 *bus-5(e2801); eEx838 [bus-5(X: 2,111,100 – 2,114,100)::gpd-2/gpd-3::GFP; unc-119(+)]*

Rescuing bicistronic construct using 1 kb *bus-5* promoter.

CB6923 *bus-6(e2728); eEx705[bus-6(V: 8,397,861 – 8,403,250)::gpd-2/gpd-3::TagRFP-T; sur-5p::GFP]*

Rescuing bicistronic 4 kb construct.

CB6957 *bus-10(e2702); eEx709[bus-10(IV: 10,901,803 – 10,908,096) :: gpd-2/gpd-2::TagRFP-T; sur-5p::GFP]*

Rescuing bicistronic 6.3 kb construct.

CB7439 *lon-1(e185) bus-22(e3108); eEx842 [bus-22 (III: 6,718,152 – 6,720,456) gpd-2/gpd-2::TagRFP-T; sur-5p::GFP]*

Rescuing bicistronic 2.3kb construct.

**Supplementary Methods**

**Detergent sensitivity assay**

SDS (sodium dodecyl sulfate) was added to NGM agar plates to final concentrations of 0, 0.04 and 0.06 %. 5 adult hermaphrodites of each strain to be tested were added to individual plates. Worms were scored for survival after two days of incubation at 25˚C.

Sensitivity ranking: % Survival at 0.04% %Survival at 0.06%

+ 100 > 60

++ < 100 < 60

+++ < 80 < 20

++++ < 20 < 20

**CRISPR/Cas9 for *bus-5***

Procedures as in Farboud and Meyer (2015).

Injection mix:

Component Final concentration

Cas9 0.96 μg/μl

TracrRNA 1.34 μg/μl

*dpy-10* gRNA 0.16 μg/μl

*dpy-10* ssDNA 13.7 ng/μl

gRNA exon 1 0.30 μg/μl

gRNA exon 5 0.30 μg/μl

KCl 24.9 mM

Hepes 7.48 mM

Cas9 was supplied by Invitrogen, Tracr RNA and gRNAs were supplied by Dharmacon.

gRNA exon 1:

5’ GACAUGCGUUCUGAUAACUGGGUUUUAGAGCUGUUUUG

gRNA exon 5:

5’GAUAUGUGGAAGACUGCUCGGGUUUUAGAGCUAUGCUGUUUUG

Wild-type BUS-5

MGSAWEEPTC VLITGGCGFI GSNYINFTFN KWKNTKFINY DKLAFGASPL

HVEKEIRESP RYKFVEAALE DQPTLIKTLQ ENEVDMVIHF AAITHVDESY

SDRIGTIQDN IISTTTLLES IVNSPYKGVK KLVHISTDEV YGDSFEDTTP

KSESASLPNP TNPYAASKAA CEMVIRSYWH SYKLPYVMVR MNNVYGPRQI

HTKLIPKFTK LALDGKPYPL MGDGLHTRSW MYVEDCSEAI TRVALEGTLG

EIYNIGTDFE MTNIELTKMI HFTVSKLLNR EPTAPTFAPI PDRPYHDRRY

YIDFSKIRNA MGWQCTTPFS EGLMKTIDYY VKLHVATARL QG*

Truncated BUS-5 encoded by *e3133*:

MGSAWEEPTC VLITGGCGFI GSNYINFTFN KWKNTKFINY DKLAFGASPL

HVEKEIRESP RYKFVEAALE DQPTLIKTLQ ENEVDMVIHF AAITHVDESY

SDRIGTIQDN IISTTTLLE**I LEILKSPTWL QTNRLLQE*** [138aa]

**SUPPLEMENTARY TABLE S1.** Source of Mutants

Gene Method Number of alleles Representative alleles

________________________________________________________________

*srf-2*  Published^1^ 1 *yj262*

Bus screen, EMS^2^ 5 *e2679*

Bus screen, *mut-7^2^* 11 *e2718*

Verde2 resistance, EMS 4 *e3100*

Million Mutation Project^5^ 2 *gk748659*

*srf-5* Published^3^ 1 *ct115*

Bus screen^2^ 1 *e2697*

Verde2 resistance, EMS 1 *e3147*

Million Mutation Project^5^ 1 *gk424525*

*bus-5* Bus screen, EMS^2^ 8 *e2801, e2985*

Verde2 res, EMS^2^ 8 *e3106, e3132*

Bah screen^4^ 1 *br19*

CRISPR/Cas-9 1 *e3133*

*bus-6* Bus screen, EMS^2^ 2 *e2691*

Bus screen, *mut-7^2^* 5 *e2728*

*bus-10* Bus screen, EMS^2^ 2 *e2702*

Bus screen, *mut-7^2^* 31 *e2714*

*bus-13* Bus screen, EMS^2^ 1 *e2710*

*bus-21* Bus screen, EMS 1 *e2992*

Bus non-comp, EMS 2 *e2997, e2998*

*bus-22* Verde2 resistance, EMS 2 *e2798, e3108*

Million Mutation Project^5^ 2 *gk850019, gk605625*

*bus-24* Verde2 resistance, EMS 1 *e3020*

Verde1 resistance, EMS 3 *e3034*

*bus-28* Million Mutation Project^5^ 1 *gk236264*

----------------------------------------------------------------------------------------------------

References: ^1^Politz *et al.* 1990, ^2^Gravato-Nobre *et al.* 2005, ^3^Link *et al.* 1992,

^4^Darby *et al.* 2007, ^5^Thompson *et al.,* 2013

**SUPPLEMENTARY TABLE S2.** *srf-2* mutation sequences

Allele Mutation Consequence

*e2697* Int 1 donor splice GT>AT   RF change, stop

*e2679* R163* (CGA>TGA Opal) RTPYETPR> RTPYETP*

*e2703* I417K (ATA>AAA) KISDGLKI > KISDGLKK

*e2718* Tc1 insertion exon XII RF change, stop

*e2734*  L256T (CTG>ACG) MTDCLLQYKE> MTDCLTQYKE

*e2767* CATA insert at M430 RF change, stop at 471

*e2777* D367E (GAT>GAA) MPVDVNA > MPVEVNA

*e2799* G37R(GGA>AGA) exon II FLIAVTILAG >FLIAVTILAR

*e2793* G335E(GGA>GAA)exon XI FKGEQKWG > FKGEQKEWE

*e2833* G335E (GGA>GAA) exon XI same as *e2793*

*e3022*  Int 2 donor splice GT> AT RF change, stop

*e3137* W125* (TGG> TAG Amber) TIPFFCKW>TIPFFCK*

*e3139* Q332* (CAA>TAA Ochre) MKFKGEQ > MKFKGE*

*e3140* W377* (TGG> TAG Amber)  NAFYHLRIW > NAFYHLRI*

*e3141* C450Y  (TGC>TAC) YYPLIEVC > YYPLIEVY

*e3142*  Q500* (CAG> TAG Amber) SYRNIYIHQ > SYRNIYIH*

*e3143* R453* (CGA> TGA Opal)  YYPLIEVCYNR > YYPLIEVCYN*

*e3144*  W334* (TGG> TGA Opal)  MKFKGEQKW > MKFKGEQK*

*e3145* R245Q (CGG>CAG)  DPNTELEFR > DPNTELEFQ

*e3146* Q133* (CAA> TAA Ochre) PYLAVGQ> PYLAVG*

*yj262* S346R (AGT>AGA) PERVDSTWIH > PRVDRTWIH

*yj33* D269N (GAT>AAT) exon VI   AAEFIVFPDPD >AAEFIVFPNPD

*yj422* D271N (GAT>AAT) exon VI   AAEFIVFPDPD >AAEFIVFPDPN

**TABLE S2 Legend: SRF-2 sequence and missense mutations**

R

MMLKSCGLIF KGKRFVRLFI FIAVCLGFLI AVTILAGLTI FDRQHNHILH

DYVARNDDIV VLSTTYYENS KSFPPNTAVI LFNSVQVFHL KYSNLNVVAE

TMQGNVEVQF KIQPVINTIP FFCKWVPYLA VGQVPEDHVL LKLSTNKIDG

MELSLRTPYE **TPRKVVACFS PLFLNERWQL LLATVEIYSH YGAFMHFYVR**

Q

**SIITDLFKLI KDNKNTRISP WSAIRIGESR AASPMFDPNT ELEFRNQASA**

T N N

**MTDCLLQYKE AAEFIVFPDP DDILVPVLGK NYYEEFTQAF KMFPTAGAVV**

E  **R**

**YNMTQTSIES SMTPALYSPI SMLASMKFKG EQKWGKLVVR PERVDSTWIH**

E

**RSYAIKEGFE QKVMPVDVNA FYHLRIWK**FP EVPTFNRSKI SNPPFFDPYH

K Y

LNATKRAIYK ISDGLKIQRK FKNRVSQGTM KTIYSRLPKV SLYYPLIEVC

YNRIFYSMKD IGTCRGPEYC NIPAFPGLRC TNVASEFVTY KSYRNIYIHQ

LISTDFEEGD NGCTL

**GT 92 domain (residues ~ 160-377) is marked in bold.**

**SUPPLEMENTARY TABLE S3**

*bus-5* mutations, sequences and class phenotypes

_____________________________________________

Class Allele Mutation

_____________________________________________

Class A: Null. Bah, BS++++, DS++++

*br19* G142E

*e2801* T94I

*e3127* E139K

*e3128*  W5stop TGA

*e3132* Q72stop TAA

*e3133* DELETION

*e3134*  R293K

Class B: Severe. Bah, BS+++, DS+++

*e2986* D297N

*e3106* R190H

Class C: Less severe. Incomplete Bah, BS +++, DS+++

*e2685*  exon1 donor: GT>AT

*e2704*  T106M

Class D: Intermediate. Non-Bah, BS+++, DS+++

*e2701* S167N

*e3110* G256R

*e3129*  A92T

*e3135*  exon4 acceptor: AG>AA

Class E: Mild. Verde2 resistant, Non-Bah, BS++, DS++

*e2686*  P163S

*e2699* D41N

Class F: Very mild. Slight Dar on Verde2, Non-Bah, BS++, DS++

*e2688* exon6 acceptor: AG>AA

*e2794*  G16S

*e2985* S99F

WT: Dar/inviable on Verde2; Non-Bah; BS+, DS+

_______________________________________________

**Legend**: BS, bleach sensitivity; DS, detergent sensitivity. BUS-5 sequence and mutations. Putative active site is underlined.

**S N**

MGSAWEEPTC VLITG**G**CGFI GSNYINFTFN KWKNTKFINY **D**KLAFGASPL

**T I F**

HVEKEIRESP RYKFVEAALE DQPTLIKTLQ ENEVDMVIHF A**A**I**T**HVDE**S**Y

**M K E**

SDRIG**T**IQDN IISTTTLLES IVNSPYKGVK KLVHISTD**E**V Y**G**DSFEDTTP

**S N H**

KSESASLPNP TN**P**YAA**S**KAA CEMVIRSYWH SYKLPYVMV**R** MNNVYGPRQI

HTKLIPKFTK LALDGKPYPL MGDGLHTRSW MYVEDCSEAI TRVALEGTLG

**R K N**

EIYNI**G**TDFE MTNIELTKMI HFTVSKLLNR EPTAPTFAPI PD**R**PYH**D**RRY

YIDFSKIRNA MGWQCTTPFS EGLMKTIDYY VKLHVATARL QG*

**SUPPLEMENTARY TABLE S4**

Strain Genotype Thrash/min

(22˚C, mean ± SD)

----------------------------------------------------------------

N2 *WT* 106 ± 4

AT6 *srf-2(yj262)* 105 ± 5

AT10 *srf-3(yj10)* 108 ± 7

CB5619 *srf-5(ct115)* 105 ± 4

CB7549 *bus-4(br4)*  102 ± 9

DC19  *bus-5(br19)*  107 ± 7

CB5613 *bus-6(e2691)* 111 ± 3

CB6177 *bus-8(e2883)*  104 ± 6

CB5617 *bus-10(e2702)*  113 ± 8

CB5635 *ptr-15(e2710)* 108 ± 5

CB7431 *bus-17(br2)* 102 ± 6

CB6037 *bus-18(e2795)* 107 ± 7

CB6338 *bus-19(e2912)* 97 ± 5

CB6794 *dhs-5(e2997)* 80 ± 4

CB6881 *bus-22(e2798)* 110 ± 11

CB6978 *bus-24(e3020)* 111 ± 7

**REPRESENTATIVE SEQUENCE DATA**

Relevant sequence data for mutations discussed in this paper are provided below. Coding sequences are indicated in upper case, introns in lower case. Mutant codons are underlined and marked in bold, with mutated bases in larger font.

Gene *srf-2* F59C6.8

Allele *yj262*

AGT>AGA. Ser346Arg. Exon 9

acagttttcacaaaaattctgaattttatggaatcactagtttaatcaattttcagGGAGAACAAAAATGGGGAAAGCTGGTAGTTCGACCAGAACGAGTCGAT**AGA**ACATGGATCCATAGATCGTATGCAATTAAAGAAGGATTTGAGCAAAAAGTGATGCCAGTTGATGTCAATGCATTTTATCATTTGAGAATATGGAAATTTCCTGAAGTTCCAACTTTCAATAGATCAAgtgagcttctgtgaagagttccatgtcatctggtagaatttttcagAAATT

Gene *srf-2* F59C6.8

Allele *e3137*

TGG>TAG Trp125Amber. Exon 3

taactaagctttcaaatagattcgaaaatgtctgaaaattcatgaagtaaacgatgtacccactcagtatttgctcaaaatccccccaatatttttttcatagGATTAACAATATTCGATCGTCAACACAATCACATTCTCCATGACTACGTGGCACGAAACGACGATATTGTGGTTTTATCGACGACATATTATGAGAATTCAAAAAGTTTTCCACCAAATACAGCTGTAATATTATTCAATTCTGTTCAAGTATTTCATTTGAAATACTCAAATTTAAACGTGGTCGCCGAGACGATGCAAGGAAACGTGGAAGTTCAGTTCAAAATTCAACCAGTAATAAATACAATTCCATTTTTCTGTAAA**TAG**GTACCCTATCTAGCTGTTGGGCAAGTACCCGAAGATCATGTTTTATTGAAATTATCAACTAATAAGATTGATGGAATGGAGgttagttgatgttaagaaaaacgaataactttacccgggacttttcctgaaaagttaccttct

Gene *srf-2* F59C6.8

Allele *e3141*

TGC>TAC Cys450Tyr. Exon 11

cgacatggatgggtgcatgtagattcaagtattactaaatatgttccataatactaaattcaatcggttcaattttaattcgaattggctcttcagATTTACTCCCGACTGCCGAAAGTTTCCCTTTACTACCCACTCATCGAAGTT**TAC**TACAATCGAATATTCTACAGTATGAAAGATATTGGAACATGTCGAGgtgagcaactcggaagcttcggcttaacctcccaattttttagGCCCAGAGTACTGTAACATTCCTGCATTCCCCGGATTACGGTGCACAAATGTGGCAAGCGAATTTGTGACTTATAAAAGTTATCGGAATATCTACATTCATCAGCTGATATCAACAGATTTTGAGGAAGGGGATAATGGATGTACTCTCTAGtcttgacg

Gene *srf-5* F54B11.10

Allele *ct115*

TGG>TGA Trp32Opal. Exon 3

attcaccgggtcacttcacttcaatATGAGAATCATCATCATCTGTCTCATTTTTCTGGCATTCTTGGTTAATCTAGTGGATAGTGTATGTAAAGCAGgttagttgccatttgtattctaatccatcaaacaaattcgatgtttgtagAGGATTACTGTCCCGGAGGT**TGA**AATGTGATGAGAAAGGCAGATGATACCCCACAGACTTGTGACGCAATGGGCGGAGTCAAATGTCAAAAACCGTATTCGTGTGTGCATTCTAGATGTGGAATGGATTTCTGTTGT

Gene *srf-5* F54B11.10

Allele *e3147*

TGT>TAT Cys70Tyr. Exon 3

AGCAGgttagttgccatttgtattctaatccatcaaacaaattcgatgtttgtagAGGATTACTGTCCCGGAGGTTGGAATGTGATGAGAAAGGCAGATGATACCCCACAGACTTGTGACGCAATGGGCGGAGTCAAATGTCAAAAACCGTATTCGTGTGTGCATTCTAGATGTGGAATGGATTTCTGT**TAT**GCTCACACTTgtaagtgcaaatatgaatgttgaaagtaaatcaaattgtgcttttccagATAAAATTGACCAATGGAAGCGACAACAAGAAAT

Gene *bus-5* F53B1.5

Allele *br19* (Class A)

GGA>GAA Gly142Glu. Exon 4

GGTACGATTCAAGACAACATTATTTCTACTACTACTCTTCTTGAGAGCATCGTCAACAGCCCATACAAGGGTGTCAAAAAGCTTGTCCATATCAGTACAGgtaaagtttggaattttcatggcttccgttttcattcttaacttttaatttttcagATGAAGTTTAC**GAA**GACTCTTTTGAAGATACCACTCCAAAGTCAGAGTCCGCATCTCTACCAAATCCAACAAATCCATATGCCGCTAGCAAGGCTGCTTGTGAGATGGTTATTAGATCCTATTGGgtaattatagtagattccttcaactattcatatatgtattttcagCACTCCTACAAACTTCCATACGTGATGGTGCGCATGAACAACGTCTATGGGCCACGCCAAATCCATACCAAGCTCATCCCAAAGTTCACCAAGCTCGCTTTGGATGGAAAACCC

Gene *bus-5* F53B1.5

Allele *e2801* (Class A)

ACC>ATC Thr94Ile. Exon 3

CACGTGGAGAAGGAGATTAGAGAGTCGCCGAGATACAAATTCGTCGAGGCTGCACTTGAGGATCAACCAACTTTGATCAAAACTCTTCAAGAGAATGAGgtaaagcctagattcgagtgctggtgtgccgacaaaatgtgaaaatgcatgatttcagGTCGATATGGTGATTCACTTTGCCGCCATC**ATC**CACGTCGACGAATCCTACAGCGACAGAATCGGTACGATTCAAGACAACATTATTTCTACTACTACTCTTCTTGAGAGCATCGTCAACAGCCCATACAAGGGTGTCAAAAAGCTTGTCCATATCAGTACAGgtaaagtttg

Gene *bus-5* F53B1.5

Allele *e3106* (Class B)

CGC>CAC Arg190His. Exon 5

TCCAACAAATCCATATGCCGCTAGCAAGGCTGCTTGTGAGATGGTTATTAGATCCTATTGGgtaattatagtagattccttcaactattcatatatgtattttcagCACTCCTACAAACTTCCATACGTGATGGTG**CAC**ATGAACAACGTCTATGGGCCACGCCAAATCCATACCAAGCTCATCCCAAAGTTCACCAAGCTCGCTTTGGATGGAAAACCCTACCCACTTATGGGAGACGGTCTTCATACTAGAAGTTGGATGTATGTGGAAGACTGCTCGGAGGCTATTACAAGAGTAGCACTTGAAGGAACACTAGGAGAAATTTACAATATTGGAACTGACTTTGAGATGACAAATATTGAACTTACGAAGATGATTCATTTCACAGTGAGCAAACTTTTGAACAGgtgagtgtaattttttcaaaacgattttgaaa

Gene *bus-5* F53B1.5

Allele *e2704* (Class C)

ACG>ATG Thr106Met. Exon 3

attcgagtgctggtgtgccgacaaaatgtgaaaatgcatgatttcagGTCGATATGGTGATTCACTTTGCCGCCATCACCCACGTCGACGAATCCTACAGCGACAGAATCGGT**ATG**ATTCAAGACAACATTATTTCTACTACTACTCTTCTTGAGAGCATCGTCAACAGCCCATACAAGGGTGTCAAAAAGCTTGTCCATATCAGTACAGgtaaagtttggaattttcatggcttccgttttcattcttaacttttaatttttcagATGAAGTTTACGGAGACTCTTTTGAAGATACCACTCCAAAGTCAGAGTCCGCATCTCTACCAAA

Gene *bus-5* F53B1.5

Allele *e2701* (Class D)

AGC>AAC Ser167Asn. Exon 4

GGTACGATTCAAGACAACATTATTTCTACTACTACTCTTCTTGAGAGCATCGTCAACAGCCCATACAAGGGTGTCAAAAAGCTTGTCCATATCAGTACAGgtaaagtttggaattttcatggcttccgttttcattcttaacttttaatttttcagATGAAGTTTACGGAGACTCTTTTGAAGATACCACTCCAAAGTCAGAGTCCGCATCTCTACCAAATCCAACAAATCCATATGCCGCT**AAC**AAGGCTGCTTGTGAGATGGTTATTAGATCCTATTGGgtaattatagtagattccttcaactattcatatatgtattttcagCACT

Gene *bus-5* F53B1.5

Allele *e2686* (Class E)

CCA>TCA Pro163Ser. Exon 3

gaattttcatggcttccgttttcattcttaacttttaatttttcagATGAAGTTTACGGAGACTCTTTTGAAGATACCACTCCAAAGTCAGAGTCCGCATCTCTACCAAATCCAACAAAT**TCA**TATGCCGCTAGCAAGGCTGCTTGTGAGATGGTTATTAGATCCTATTGGgtaattatagtagattccttcaactattcatatatgtattttcagCACTCCTACAAACTTCCATACGTGATGGTGCGCATGAACAACGTCTATGGGCCACGCCAAATCCATACCAAGCTCATCCCAAAGTTCACCAAGCTCGCTTTGGATGGAAAACCCTACCCACTTATGGGAGACGGTCTTCATACTAGAAGTTGGATGTATGTGGAAGACTGCTCGGAGGCTATTACAAGAGTAGCACTTGAAGGAACACTAGGAGAAATTTACAA

Gene *bus-5* F53B1.5

Allele *e2699* (Class E)

GAT>AAT Asp41Asn. Exon 2

tccgccgccaaaaagagATGGGTTCCGCGTGGGAAGAACCAACATGCGTTCTGATAACTGGCGGCTGTGGATTCATTGGGTCCAACTACATAAACTTTACCTTCAATAAATGGAAAAACACAAAgtaacacaacactcgaaatctctcgagtatgtataataattgtacttcagaTTCATTAACTAC**AAT**AAATTGGCTTTTGGAGCTTCTCCGCTGCACGTGGAGAAGGAGATTAGAGAGTCGCCGAGATACAAATTCGTCGAGGCTGCACTTGAGGATCAACCAACTTTGATCAAAACTCTTCAAGAGAATGAGgtaaagcctag

Gene *bus-5* F53B1.5

Allele *e2794* (Class F)

GGC>AGC Gly16Ser. Exon 1

agatccgccgccaaaaagagATGGGTTCCGCGTGGGAAGAACCAACATGCGTTCTGATAACTGGC**AGC**TGTGGATTCATTGGGTCCAACTACATAAACTTTACCTTCAATAAATGGAAAAACACAAAgtaacacaacactcgaaatctctcgagtatgtataataattgtacttcagaTTCATTAACTACGATAAATTGGCTTTTGGAGCTTCTCCGCTGCACGTGGAGAAGGAGATTAGAGAGTCGCCGAGATACAAATTCGTCGAGGCTGCACTTGAGGATCAACCAACTTTGATCAAAACTCTTCAAGAGAATGAGgtaaagcctagattcgagtgctggtgtgccgacaaaatgtgaaaatgcatgatttcagGTCGATATGGTGATTCACTTTGCCGCCATCACCCACGTCGACGAATCCTACAGCGACAGAATC

Gene *bus-5* F53B1.5

Allele *e2985* (Class F)

TCC>TTC Ser99Phe. Exon 3

CACGTGGAGAAGGAGATTAGAGAGTCGCCGAGATACAAATTCGTCGAGGCTGCACTTGAGGATCAACCAACTTTGATCAAAACTCTTCAAGAGAATGAGgtaaagcctagattcgagtgctggtgtgccgacaaaatgtgaaaatgcatgatttcagGTCGATATGGTGATTCACTTTGCCGCCATCACCCACGTCGACGAA**TTC**TACAGCGACAGAATCGGTACGATTCAAGACAACATTATTTCTACTACTACTCTTCTTGAGAGCATCGTCAACAGCCCATACAAGGGTGTCAAAAAGCTTGTCCATATCAGTACAGgtaaagtttggaattttcatggcttccgttttcattcttaacttttaatttttcagATGAAGTTTACGGAGACTCTTTTGAAGATACCACTCCAAAGTCAGAGTCCGCATCTCTACCAAA

Gene *bus-6* F52E1.9

Allele *e2691*

G…GT>G…AT Gly48Asp. Exon 3

GCCTCCTGGACAAGCATTTCCTTTTGAACCATTGCCACCATATCCAGCA**G**gtttgtttctttaaaagaaaacttttacgtataacattttctagctgcgactagaatcagattttttcaatactcgcgaactggtagaacagttttttttccactaaactattaataatttcgctatcttttcag**AT**CCAAATCTCTCAAAAATCCGCTACCCCCGTGAAGTGACCTGTCCCCGTTACGTATGTCCACACTGTGACGAACAATTCCTATTCCACTCAACAAACGGACTCCTCACTTGCCC

Gene *bus-6* F52E1.9

Alleles *e2759, e2790*

TGT>T//GT Cys78Tc1. Exon 3

gcgaactggtagaacagttttttttccactaaactattaataatttcgctatcttttcagGTCCAAATCTCTCAAAAATCCGCTACCCCCGTGAAGTGACCTGTCCCCGTTACGTA**T**/cagtgctgg… (Tc1)/

**GT**CCACACTGTGACGAACAATTCCTATTCCACTCAACAAACGGACTCCTCACTTGCCCATTTTGCTACACATCAATTGCAATTGGCACTTATgtgagtttgacggttactgtagcagtaattttgtaactaatatttcagAATCGCAAA

Gene *bus-10* ZK596.3

Allele *e2702*

TGG>TGA. Trp119Opal. Exon 3

aaaaattaccgaaattcaaagtttgtttcaagGTTTATCCAAATGGAACCAAATACTATGAACTAGAACGACCAGATGGAACAATATTTGTGGGAGGACTTGGACCATGGTTTGATCGTCTTTTGATGGGAGAATGGAGATATTGGTATGGAAATACATTTTACAATTATTGTAGAgtgagtttcgtttttctaaaaaatagtttactaaataagaatatttagCCTCGTGATCCTCCAAGAACAGAA**TGA**ATGACATCGATTTTACGGTCAATGAATCACTTGGTTATAGTACAGgtttgttcaattgtcaaatcaattaacggaaactggaacaatgtatggttgttaggggtttttgtcgtctagaaaggcacacaaacacctgccttcgtgcctg

Gene *bus-10* ZK596.3

Allele *e2715* (3.2 kb deletion allele)

5’ AAGTGTGATAATTAAAGTAGT//TATTTGTTGTATTTACTTC 3’

Gene *bus-10* ZK596.3

Allele *e2737* (5.8 kb deletion allele)

5’ CTCAAATTACTTTAATTTA//CCCACGACAAGGAACCGTTT 3’

Gene *ptr-15*

Allele *e2710*

GAA>AAA Glu364Lys. Exon 7

ATGgtaattatttttgatacggataattcgaaaattttcaaacaaaatcacttaatatcctttcagTATGACTGACAATTGGGTGACATCAAAACCAATTGAAGCCATGATCGGAATTCTTGTCTCTTCAATGGCAATTGTATCAGCTGGTGGATTGTTATTTGCTCTAGGAGTACCATTTATCAATCAGGTCACTGTTATGCCATTCATCGCCTTGGCTATTGGAGTTGATGACGTTTATGTTATGCTGGGTGCTTGGCAAGATACCAGAAGAACTCTCGATGCCAAAAAAAGAATGGGGTTAGCTCTGGAG**AAA**GCTGGAAGTGCAATCACAGTTACTTCACTTACTTCAGTGCTCTCTTTTGGTATTGGAACATACTCAACAACTCCTGCTATAGCAATTTTTTGTAAATTCATTGCGTTGGCTATCATGTTTGATTGGTTTTACCAGCTAACGTTTTTTGCGGCAGTTATGGCTATGGGAGCAAAGAGAGAAGCAGCTGGATATCATTGCGTGTTTGTCTGGAAACGTTGTGATCGAGAAGAAATTGCTAAGGCTAAAAACGAAAATGCAATTTCACCAACAAGATACTTTTTTGAAAACATATTCGCGCCATTCATCTGCCGCCCATCTGTTAGATTCTGCATGgtcagttaaaaaaagtgtaactctttaaaaatttttcgcgaaatctttcag

Gene *bus-22* F37A4.3

Allele *e2798*

CGA>TGA Arg16Opal. Exon 1

tgatttgacggttttttgctgttttccagttaaattatgcacttattagagaagatatctgaATGATGGAACCAAAATCTATATTTTTATTGGGCTTGTTACTATTC**TGA**GTTGGGAAACTTATGAGAAACGATAAACTCGCATGGAGCGCGGAATACGAAGCAAAgtaatttcatatttttcattaaaatcaaaactatagtagttaactataaataattgttttaatttacagAAAATTCAAGTTTTATCACAATACATCTTTATTTCTAACTTTACAATCTACGGGTCCTGAGATGg

Gene *bus-21/dhs-5* F56D1.5

Allele *e2992*

TGT>TAT Cys239Tyr. Exon 4

tttgacgggaaaatcccaagaaacattgaatatatttattcaaagctgccattcagTGAACAGCGTTGGAACTGGTCGCGACAATTTGGAAAGATATGGAGACAATCCAGATGAGGACACTCAAATATTGAGAGTAAATGGAATGGGTGCAGCCGAGTTCCTATCATGTGTGCTCCCACCAATGGAGAAATCTGGAGGTGGACAGATTGTTGTGCTCTCGTCTTCACAAGGAGTACGGCCAATACCCATGCTTGCGGCTTAT**TAT**GCAACAAAGGCTTTGATGACGTTCTTGTGCGAATCGATTGATCGCGAGTACAGTACAATCAATGTTCAAACTTTGATCCCTGCATTGGTCGCCACAAAAATGACCTACTACACGgtaagcagttatttgcgaagttcttagaaaattaatttacaatcttatttccagAAAGGATCTACATTCGTTGTGACACCAGAAAACTTCTGCCACCAGGCGGTTGGCTCAATTGGATTGACGAAGAAGACTGCTGGATGTTTGAACCATGAGCTTCAAATGCTCGGATTCCACCTGTTCCCATGGACTATTCTCAAGTACCTTATCATGCCAATCTACTATCATCAGAGAAAACGTGTGACAGAATTGCACAATACGAGCAATAACCCAGAACAGGAAATTTCACTTCAAGAATTGAATGAGGA

Allele *e2997*

GGA>GAA Gly120Glu. Exon 2

tgatataaaactcataggtgtgaaaacaagttgcgttcaggttactgtagaggtatggtagagatataaacttgattttttcagAACATTCTTTGCATTCCTGAAAGCAATCTTCATCTACACAATTGCACCTCTCTTCTACAAACCAAATCTGGAACAGTACCAACACAGATGGACAGTCGTCTCCGGTGGAACCGATGGAATTGGAAAAGCTTACACATTGGAACTTGCAAAGCGAGGTCTCCGGAAGTTTGTACTTATT**GAA**AGAAATCCAAAGAAACTTGATTCGGTTAAATCAGAAATTGgtaagttgttttattattatttagtattgttaatttatttaattttcagAGGAGAAGCACTCGGACGCTCAAATCAAGACTTTTGTGTTTGACTTTGGTAGTGGCGACTTCTCAAGCCTTCGCGATTACATCAGCGACATCGATGTTGGATTTGTCGgtaagaagttgggtgaaaaggggaataatatgagggaatgtagggcagatt

Gene *bus-24* Y11D7A.9

Allele *e3020*

TCA>TTA Ser88Leu. Exon 3

GTAACTATAATTGGCAGTGTGGTgttagtttatttttatttttgagtttattttaggattcaaaaaaatccaaatttttcagCGGGCTCGTCTTCCC**TTA**CTCTCCAGAATTATTAATTTACCAGTTGAACGTACGTTTTGGCAACTTTTTCTCTTGTTTCATGTGCCTATTCGAGTTGTTGAGCTGATCACAGGATgtaagtttttcgttttgttaaaacattgaatcatgtttgaaatatttttttcgaaaaaattttgtttgaaatttcttaaaatatagattcattgttgagaaattgaccagtttttttctagTTTCCCGATACAAACGAATGAGAAATGTGAACTA

Allele *e3034*

GGA>GAA Gly120Glu. Exon 3

AGTAACTATAATTGGCAGTGTGGTgttagtttatttttatttttgagtttattttaggattcaaaaaaatccaaatttttcagCGGGCTCGTCTTCCCTCACTCTCCAGAATTATTAATTTACCAGTTGAACGTACGTTTTGGCAACTTTTTCTCTTGTTTCATGTGCCTATTCGAGTTGTTGAGCTGATCACA**GAA**TgtaagtttttcgttttgttaaaacattgaatcatgtttgaaatatttttttcgaaaaaattttgtttgaaatttcttaaaatatagattcattgttgagaaattgaccagtttttttctagTTTCCCGATACAAACGAATGAGAAATGTGAACTA

**Supplementary Literature Cited**

Darby C, Chakraborti A, Politz SM, Daniels CC, Tan L, *et al*. 2007.

*Caenorhabditis elegans* mutants resistant to attachment of Yersinia biofilms. Genetics. 176:221-230.

Farboud J, Meyer BJ. 2015.

Dramatic enhancement of genome editing by CRISPR/Cas9 through improved guide RNA design. Genetics 199:959-971.

Gravato-Nobre MJ, Nicholas HR, Nijland R, O'Rourke, D, Whittington D *et al*. 2005.

Multiple genes affect sensitivity of *Caenorhabditis elegans* to the bacterial pathogen *Microbacterium nematophilum*. Genetics. 171:1033-1045.

Link CD, [Silverman MA](https://wormbase.org/resources/person/WBPerson1462), Breen M, [Watt KE](https://wormbase.org/search/all/Watt%20KE), [Dames SA](https://wormbase.org/resources/person/WBPerson900). 1992.

Characterization of *Caenorhabditis elegans* lectin-binding mutants. Genetics. 131:867-881.

Politz SM, Philipp M, Estevez M, O'Brien PJ, Chin KJ. 1990.

Genes that can be mutated to unmask hidden antigenic determinants in the cuticle of the nematode *Caenorhabditis elegans*. Proc Natl Acad Sci USA. 87: 2901-2905.

Thompson O, [Edgley M](https://wormbase.org/resources/person/WBPerson154), Strasbourger P, [Flibotte S](https://wormbase.org/resources/person/WBPerson9327), Ewing B, *et al.* 2013.

The million mutation project: a new approach to genetics in *Caenorhabditis elegans.* Genome Res. 23:1749-1762.
